# In vivo metallophilic self-assembly of a light-activated anticancer drug

**DOI:** 10.1038/s41557-023-01199-w

**Published:** 2023-05-11

**Authors:** Xue-Quan Zhou, Peiyuan Wang, Vadde Ramu, Liyan Zhang, Suhua Jiang, Xuezhao Li, Selda Abyar, Panagiota Papadopoulou, Yang Shao, Ludovic Bretin, Maxime A. Siegler, Francesco Buda, Alexander Kros, Jiangli Fan, Xiaojun Peng, Wen Sun, Sylvestre Bonnet

**Affiliations:** 1 State Key Laboratory of Fine Chemicals, Dalian University of Technology, Dalian, People’s Republic of China; 2 Leiden Institute of Chemistry, Universiteit Leiden, Leiden, the Netherlands; 3 Department of Molecular Biochemistry and Pharmacology, Istituto di Ricerche Farmacologiche Mario Negri IRCCS, Milan, Italy; 4 Key Laboratory of Design and Assembly of Functional Nanostructures, Fujian Institute of Research on the Structure of Matter, Chinese Academy of Sciences, Fuzhou, People’s Republic of China; 5 Department of Chemistry, Johns Hopkins University, Baltimore, MD USA

**Keywords:** Photobiology, Drug delivery, Bioinorganic chemistry, Melanoma

## Abstract

Self-assembling molecular drugs combine the easy preparation typical of small-molecule chemotherapy and the tumour-targeting properties of drug–nanoparticle conjugates. However, they require a supramolecular interaction that survives the complex environment of a living animal. Here we report that the metallophilic interaction between cyclometalated palladium complexes generates supramolecular nanostructures in living mice that have a long circulation time (over 12 h) and efficient tumour accumulation rate (up to 10.2% of the injected dose per gram) in a skin melanoma tumour model. Green light activation leads to efficient tumour destruction due to the type I photodynamic effect generated by the self-assembled palladium complexes, as demonstrated in vitro by an up to 96-fold cytotoxicity increase upon irradiation. This work demonstrates that metallophilic interactions are well suited to generating stable supramolecular nanotherapeutics in vivo with exceptional tumour-targeting properties.

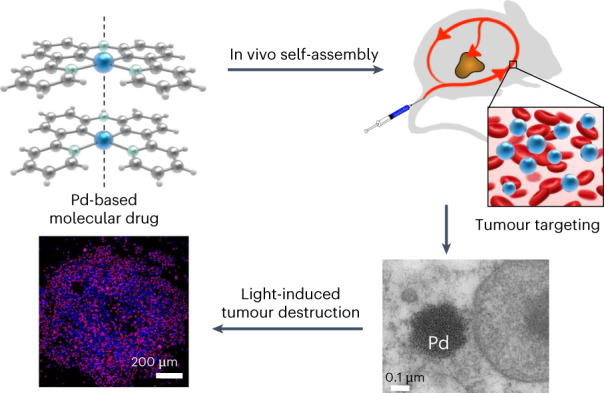

## Abstract (web summary)

The metallophilic interaction between cyclometalated palladium complexes can facilitate supramolecular nanostructure formation in living mice, providing a phototoxic prodrug with a long circulation time and high tumour-targeting efficiency. Upon green light irradiation, this palladium-based drug destroys solid tumours, leaving non-irradiated organs intact.

## Main

Curing cancer is one of the toughest challenges of modern medicine^1^, and chemotherapy occupies the front line of cancer treatment^2–4^. Many chemotherapy agents are well-defined small molecules that typically lead to nonspecific delivery, rapid blood clearance and low accumulation in tumours, altogether generating severe side effects for patients with cancer^5,6^. To overcome these limitations, they have been conjugated to tumour-targeting nanocarriers, either covalently or supramolecularly, which in principle enhances drug delivery to the tumour^7–18^. However, many nanocarriers show comparatively low drug-loading capacity (typically <20%)^19,20^, while the resulting tumour accumulation remains disappointingly low: recent studies showed that a median 0.7% of the administered nanoformulated drug dose ends up in solid tumours^20,21^. In addition, achieving the reproducible preparation of drug–nanoparticle conjugates is often challenging, which restricts the clinical applications of nanodrugs.

Drug self-delivery systems (DSDSs) may solve this issue. They consist of small-molecule drugs that self-assemble into nanostructures without the assistance of dedicated nanocarriers^22^. These systems combine the easy preparation of small molecules and the tumour-targeting properties of nanoconjugates, achieving high drug-loading efficiency^2^. They also require supramolecular forces that hold in the complex biological environment of a living animal. DSDSs proposed to date rely on a combination of hydrophobic forces, hydrogen bonding and/or metal–ligand coordination. The so-called metallophilic interaction is another kind of supramolecular interaction occurring specifically between d^8^ or d^10^ metal centres. It is well known in optoelectronics and material science^23^ for its ability to modify the photophysical and photochemical properties of metal compounds. It has also been proposed for biological applications, but only in vitro demonstrations have been made^24^. In this Article, we demonstrate with a light-activated DSDS that the metallophilic interaction survives blood circulation in living mice, leading to self-assembled nanoparticles that show outstanding tumour-targeting and phototherapeutic properties in a human skin melanoma xenograft.

## Results and discussion

### Synthesis and characterization of a light-sensitive DSDS

**Fig. 1:**
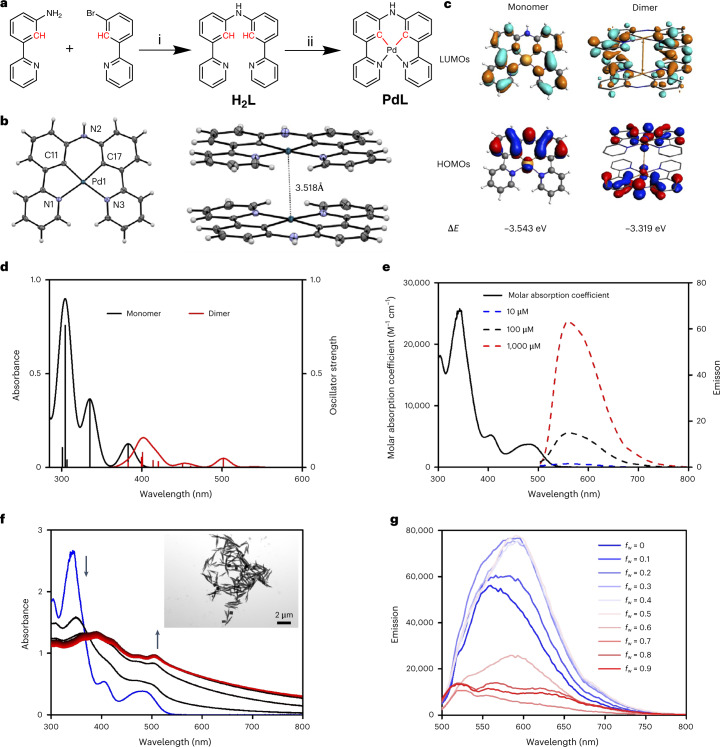
Synthesis, crystal structure, DFT calculation and photophysical properties of PdL. **a**, Synthesis of **H**_**2**_**L** and **PdL** ((i) Pd(dba)_2_, KO*t*-Bu, BINAP and toluene at 95 °C under N_2_ for 72 h with a yield of 67%; (ii) Pd(OAc)_2_ and CH_3_COOH at 135 °C for 24 h with a yield of 56%). **b**, Displacement ellipsoid plot (50% probability level) of **PdL** and its stacking structure at 110(2) K. **c**, DFT calculation of HOMOs (bottom) and LUMOs (top) of **PdL** as a monomer or dimer (calculated Pd⋯Pd distance = 3.26 Å). Occupied orbitals (HOMOs) have red and blue lobes, whereas unoccupied orbitals (LUMOs) have brown and cyan lobes. Element colour code: blue = N, grey = C, brown = Pd and white = H. **d**, TDDFT-calculated spectra of **PdL** as a monomer (black line) or dimer (red line). Level of theory: TDDFT/PBE0/TZP/COSMO (in methanol). **e**, Absorption spectrum (black solid line) and emission spectra of **PdL** in pure DMSO solution at different concentrations (blue dashed line = 10 µM; black dashed line = 100 µM; red dashed line = 1,000 µM; excitation = 419 nm). **f**, Time evolution of the absorption spectra of H_2_O/DMSO solutions (100 µM; *f*_w_ = *V*_water_/*V*_total_ = 0.9) of **PdL** at 298 K for 30 min (30-s interval; the colours of the spectra change from black (0 min) to red (30 min); the blue line is the absorbance spectrum of **PdL** (100 µM) in pure DMSO. Inset, TEM images of nanostructures of **PdL** in H_2_O/DMSO solution (100 µM; *f*_w_ = *V*_water_/*V*_total_ = 0.9; scale bar, 2 µm). **g**, Emission spectra of **PdL** (100 µM) in H_2_O/DMSO mixtures with different volumetric ratios *f*_w_. Source data

As in the padeliporfin photosensitizer recently approved for clinical photodynamic therapy (PDT)^25^, the **PdL** small molecule (Fig. 1a) contains a palladium(ii) metal centre. In contrast with padeliporfin, however, **PdL** is a bis-cyclometalated palladium compound characterized by the presence of two Pd–C covalent bonds (see Fig. 1b and full characterization in Supplementary Figs. 3 and 4 and Supplementary Table 1). Its X-ray structure (Fig. 1b) shows head-to-tail dimers with a short interplanar distance of 3.4 Å and a short Pd⋯Pd distance of 3.518 Å, characteristic of metallophilic interactions. A density functional theory (DFT) model of the supramolecular dimer converged at a Pd⋯Pd distance of 3.26 Å (Fig. 1c), matching well with that observed in the crystal. The metallophilic interaction derives from the hybridization of both palladium *d*_4z_^2^ orbitals and π orbitals of the aromatic ligand in the highest occupied molecular orbital (HOMO) of the dimer. A dimerization energy of −40.0 kcal mol^−1^ was found for the dimer [**PdL**]_2_, while that of the ligand dimer [**H**_**2**_**L**]_2_ (where **H**_**2**_**L** is (bis(3-(pyridin-2-yl)phenyl)amine)) was −33.7 kcal mol^−1^. As the ligand H_2_L can only dimerize via π–π interactions, these calculations suggest that the overlap of the palladium *d*_4z_^2^ orbitals contributed ~16% to the supramolecular interaction between two **PdL** molecules in the dimer, while π–π stacking contributed ~84%. Time-dependent DFT (TDDFT) calculations at the same degree of theory further confirmed the decrease in the gap between the HOMO and the lowest unoccupied molecular orbital (LUMO) induced by supramolecular dimerization, with a bathochromic shift of the lowest-energy absorption band from 383 nm for the monomer to 502 nm for the dimer (Fig. 1d).

When dissolved in dimethyl sulfoxide (DMSO) in air, **PdL** showed a low emission quantum yield (*φ*_p_ = 0.0016) with a short lifetime (*τ* = 0.295 µs) (Fig. 1e and Supplementary Table 6). Under degassed conditions, the emission quantum yield and lifetime increased by 1–2 orders of magnitude (0.07 and 29.6 μs, respectively), demonstrating the triplet character of the excited state of **PdL**. The corresponding photoluminescence quenching efficiency was ~98%, which suggested that ^3^PdL* efficiently interacts with molecular oxygen. Transient spectra under aerated and degassed conditions (Supplementary Fig. 5) demonstrated that the initially formed singlet excited state ^1^PdL* performs fast intersystem crossing to ^3^PdL* (rate = 34.5 ns^−1^), which then reacts with O_2_ via electron transfer to generate a **PdL**^+^ cation radical. This result strongly suggested that **PdL** may engage in PDT type I reactivity to generate superoxide radicals (O_2_^•−^) by electron transfer.

In pure DMSO, **PdL** (100 μM) was mostly monomeric (Supplementary Fig. 6a) and minute variations of the ultraviolet–visible spectrum with temperature tentatively suggested low-affinity dimerization with a dimerization constant of 10^3^–10^4^ M^−1^ (Supplementary Fig. 6b,c and Supplementary Table 7)^26,27^. In contrast, in a DMSO/H_2_O 1/9 mixture (100 µM), a rapid (<1 min) increase in the baseline and the generation of a new absorbance peak at 504 nm were observed (Fig. 1f), which are typical for metal–metal-to-ligand charge transfer excited states induced by Pd⋯Pd interactions^28^ and altogether suggest efficient and fast self-assembly. This hypothesis was confirmed by transmission electron microscopy (TEM) images showing nanorods and nanocubes (Fig. 1f, insert). Usually, the formation of Pd⋯Pd supramolecular bonds is accompanied by a long-wavelength emission peak^28^, and indeed an increase in the H_2_O content in DMSO/H_2_O mixtures (*f*_w_ = *V*_water_/*V*_total_ is the ratio of the water volume *V*_water_ and the total volume of the solution *V*_total_) led to a gradual replacement of the monomeric emission peak at 564 nm (as observed in pure DMSO; *f*_w_ = 0) by new emission maxima at 593 nm (*f*_w_ = 0.5) and finally 610–670 nm (*f*_w_ = 0.9) concomitant with the formation of a precipitate (Fig. 1g). In tetrahydrofuran/H_2_O solutions, similar self-assembly was observed, albeit with slower polymerization rates and different morphologies (Supplementary Figs. 7 and 8). The two series of self-assembled nanoparticles showed different powder X-ray diffraction patterns that were also different from the pattern calculated from the crystal structure or obtained from a bulk solid sample of **PdL** (Supplementary Fig. 9). This result indicated that the four types of materials are different polymorphs. Overall, **PdL** appeared to self-assemble readily in the presence of water, although the resulting self-assembly depended entirely on the detailed composition of the solvent it was dissolved in.

**Fig. 2:**
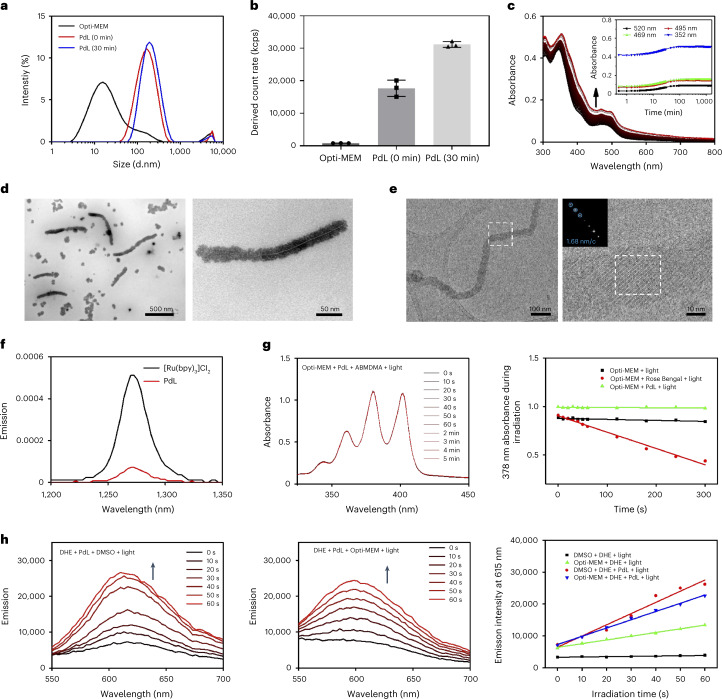
Self-assembly and aggregation nanostructure of PdL in cell medium. **a**, Size distribution of Opti-MEM complete medium and its **PdL** (25 µM) solution at 0 min (red line) or 30 min (blue line) according to DLS analysis at room temperature. d.nm, particle diameter in nanometer. **b**, DLS-derived count rate of **PdL** solution in Opti-MEM complete medium for 0 and 30 min. The data represent means ± s.d. of three replicates. kcps, kilocounts (of photons) per second. **c**, Observed absorbance spectra of **PdL** (25 µM) in Opti-MEM complete medium over 24 h (30-s intervals for the first 30 min and 15-min intervals for the remaining 23.5 h). **d**,**e**, TEM (**d**) and cryo-EM images (**e**) of samples prepared from an Opti-MEM complete medium solution of **PdL** (25 µM) at room temperature. Insert in **e** shows the Fourier transform of the atomically resolved area highlighted by a dashed box. nm/c, nanometer per cycle. **f**, Singlet oxygen emission spectra of [Ru(bpy)_3_]Cl_2_ (black) and **PdL** (red) in CD_3_OD irradiated with blue light (*λ*_ex_ = 450 nm; 50 mW; 0.4 W cm^−2^). **g**, Time evolution of the absorption spectrum (left) and of the absorbance at 378 nm (right) of a 9,10-anthracenediyl-bis(methylene)dimalonic acid (ABMDMA) Opti-MEM complete solution (100 µM) in the presence of PdL (25 µM) under green light irradiation (515 nm; 2.0 mW) over 5 min. **h**, Time evolution of the emission spectrum (left, middle) and of the emission intensity at 615 nm (right) of a DHE solution in DMSO (left) or Opti-MEM complete (middle) in the presence or absence of PdL (25 μM) under green light irradiation (515 nm; 2.0 mW) over 60 s. Source data

The self-assembly of **PdL** was then studied in a cell-growing medium called Opti-MEM complete that contained 2.5 vol% foetal calf serum. At 25 µM, aggregation immediately occurred, as shown by a hydrodynamic diameter of approximately 164 nm determined by dynamic light scattering (DLS; Fig. 2a). After 30 min, the maximum hydrodynamic diameter had only slightly shifted to 190 nm, but the number of particles had increased significantly (Fig. 2b). The absorption of the solution (Fig. 2c) showed a gradual baseline increase during the first 2 h, which is characteristic of light scattering by nanoparticles, to remain constant over 24 h. The main nanostructures observed by TEM in the medium were nanodots (Fig. 2d), but these nanodots self-assembled as regular nanofibres that gradually lengthened. Cryogenic electron microscopy (Cryo-EM) imaging confirmed the formation of nanofibres in such medium, characterized by a well-ordered structure at a repeating distance of ~1.68 nm in the Fourier transform image (Fig. 2e). As for the monomer in DMSO, in medium the photoluminescence lifetime of aggregates of **PdL** was dramatically quenched by O_2_ from 83.5 to 0.058 μs (Supplementary Fig. 5g,h), suggesting that the aggregates reacted even faster with O_2_ than molecular **PdL** (in DMSO). Also, the different shapes of the nanorods in Fig. 2d compared with those in DMSO/H_2_O (Fig. 1f) or tetrahydrofuran/H_2_O mixtures (Supplementary Fig. 7d) suggested that nanorod formation may involve proteins in the medium. Hence, we determined the protein content of the nanoaggregates formed in cell medium via protein gel and the commercial Pierce BCA Protein Assay Kit. The results (Supplementary Fig. 10) indicated that the amount of protein involved in the stabilization of the nanoparticles in medium was very low, and that the different shapes observed under such conditions must be due to other factors, such as compound concentration, solvent polarity, pH, ionic force, the presence of salts, viscosity, and so on. Overall, in cell-growing medium, DLS, electron microscopy, ultraviolet–visible spectroscopy and protein determination demonstrated the time-dependent self-assembly of **PdL** into nanorods and nanoparticles, which, considering DFT and crystal structure analysis, must at least in part be a result of the metallophilic Pd⋯Pd interaction.

As the next step, the influence of self-assembly on the photochemical properties of **PdL** was considered. Photodynamic effects may occur either via a type I mechanism (electron transfer) or a type II (energy transfer) mechanism^29^. Direct detection of the near-infrared emission peak of ^1^O_2_ at 1,270 nm under blue light irradiation (450 nm) was only possible in CD_3_OD and hence for the **PdL** monomer. The corresponding ^1^O_2_ generation quantum yield was very low (*φ*_Δ_ = 0.09; Fig. 2f and Supplementary Table 6). In Opti-MEM medium, and hence for the self-assembled form of **PdL** (25 µM), indirect ^1^O_2_ detection using the chemoselective chemical probe 9,10-anthracenediyl-bis(methylene)dimalonic acid showed no decrease in the absorbance band at 378 nm upon green light irradiation, characteristic of the ^1^O_2_ adduct (Fig. 2g)^30^, indicating negligible ^1^O_2_ generation (*φ*_Δ_ = 0.04; Supplementary Fig. 8)^31^. Overall, **PdL** is a poor PDT type II sensitizer, both as a monomer in methanol and as aggregates in medium. In contrast, type I PDT sensitizers can be characterized by the initial generation of superoxide radicals (O_2_^•−^), which can further generate other reactive oxygen species (ROS), such as HO^•^ or H_2_O_2_ (ref. ^32^). When a DMSO or Opti-MEM solution of **PdL** (25 µM) was irradiated with green light in the presence of dihydroethidium (DHE)—a chemoselective chemical probe for superoxide—the oxidation product 2-hydroxyethidium was produced efficiently, as shown by its emission at 590–620 nm (Fig. 2h and Supplementary Fig. 9)^33^. These results clearly demonstrated that **PdL** is capable of photochemically generating superoxide both in the monomeric and aggregated states, which suggested that as a light-activated DSDS it may behave as a PDT type I photosensitizer, generating its photodynamic effect by electron transfer.

### Biological properties in vitro and in vivo

**Fig. 3:**
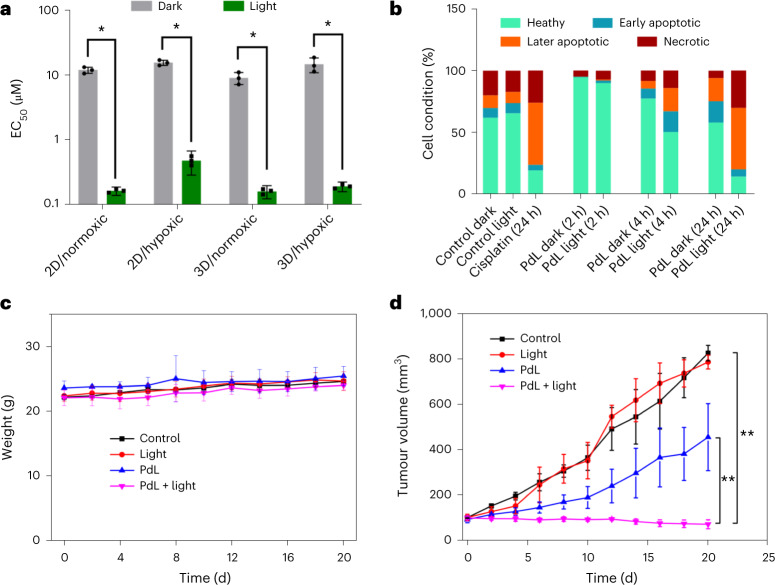
In vitro and in vivo anticancer properties of PdL. **a**, EC_50_ values of **PdL** in A375 2D monolayer and 3D spheroid cancer cells incubated either in the dark or upon green light irradiation (13 J cm^−2^) and under normoxic or hypoxic conditions. The data points represent averages (*n* = 3) with 95% confidence intervals. Statistical significance was determined by two-way analysis of variance (ANOVA) (*P < 0.05). **b**, Flow cytometry quantification of healthy, early apoptotic, later apoptotic and necrotic A375 cells after treatment with **PdL** (2 µM) in the dark or with green light irradiation over a time gradient (2, 4 and 24 h). Cisplatin (7.5 µM; 24 h) was used as a positive control. **c**, Time evolution of mouse weight up to 20 d post-treatment. **d**, A375 tumour growth inhibition in different groups of mice treated by tail intravenous injection. The data represent means ± s.d. of three replicates. Statistical significance was determined by two-way ANOVA (**P < 0.05). Light irradiation conditions: 520 nm, 100 mW cm^−2^, 10 min and 60 J cm^−2^. Dose: 2.1 µmol kg^−1^, 420 µM, 100 µl DMEM (10% FBS) and 0.9 mg kg^−1^. Source data

**Extended Data Fig. 1:**
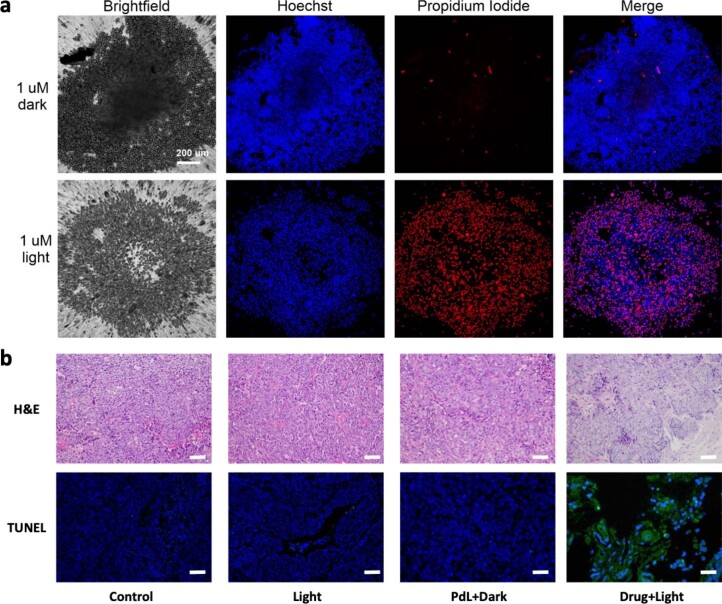
Confocal images of 3D-normoxic A375 spheroids, H&E and TUNEL staining assay of mice tumor slices after treated with PdL under dark or green light irradiation. (a) Z-stack overlapped confocal images of 3D-normoxic A375 spheroids (scale bar 200 µm) in the dark or green light irradiation, with Hoechst 33342/propidium iodide double staining after treatment with **PdL** (1 µM) for 72 h. (b) H&E and TUNEL staining assay of tumor slices of mice in different groups at day 5 (scale bar 200 μm for top and 400 μm for bottom).

Considering the good absorption of **PdL** at 520 nm (*ε* = 915 M^−1^ cm^−1^ in DMSO) and its PDT type I properties, its cytotoxicity was evaluated first in vitro using two-dimensional (2D) monolayers of lung carcinoma (A549), epidermoid carcinoma (A431) and skin melanoma (A375) cell lines, both in the dark and under green light irradiation. **PdL** showed moderate dark cytotoxicity (half-maximal effective concentration (EC_50_) > 10 µM) for the three cancer cell lines under normoxic (21% O_2_) and hypoxic (1% O_2_) conditions (Supplementary Table 8). In contrast, upon green light irradiation (520 nm; 13 J cm^−2^) under normoxia and hypoxia, **PdL** exhibited high phototoxicity with submicromolar EC_50_ and high photoindices (EC_50,dark_/EC_50,light_ = 32–72; Fig. 3a, Supplementary Fig. 10 and Supplementary Table 8), thus demonstrating outstanding PDT efficacy even at low dioxygen concentrations. Clearly, at the concentrations used (0.5 and 2 µM), **PdL** showed no or limited cell death under dark conditions, as determined by annexin V/propidium iodide double staining experiments (Fig. 3b and Supplementary Fig. 11). In the light-irradiated group, no toxicity was observed after 2 h, but after 4 h and 24 h, the numbers of apoptotic and necrotic cells had increased, suggesting that **PdL** induced cancer cell death 4 h after irradiation via both cell death mechanisms. The cytotoxicity of **PdL** in 3D multicellular tumour spheroid models (A549 and A375), which better mimic the physical penetration of light and drugs in three dimensions^34^, was nearly 100-fold higher under light irradiation (EC_50_ = ~0.20 µM) than under dark conditions (EC_50_ > 25 µM), while light activation was accompanied by visible collapse of the spheroid cores and dramatic shrinkage of the spheroid diameters (Extended Data Fig. 1a, Supplementary Fig. 12 and Supplementary Table 8). A further Hoechst 33342/propidium iodide double staining experiment was carried out to compare the morphology and health status of A375 spheroids after treatment. The red fluorescence of propidium iodide increased in the green light-irradiated group compared with the dark group (Extended Data Fig. 1a for 1 µM), confirming drug penetration and light-induced cell killing by membrane disruption in 3D environments.

To explain the phototoxicity observed in vitro, a few mechanistic studies were undertaken. First, the intracellular ROS (as demonstrated with the non-selective dichlorodihydrofluorescein diacetate (DCFDA) probe), and especially superoxide levels (as demonstrated with the DHE probe), were found to be significantly increased after light irradiation in the presence of **PdL** (Supplementary Fig. 16), while the glutathione levels decreased (Supplementary Fig. 17). These results confirmed the conclusion of photophysical studies—that electron transfer (PDT type I) is probably the main source of the phototoxicity of **PdL** aggregates in cells. A cellular fractionation experiment was then realized to see where the palladium was mainly located (Supplementary Fig. 18); clearly, **PdL** accumulated in the membrane fraction (which includes cell, mitochondria and lysosome membranes) and the nuclei (Supplementary Fig. 18). This result was consistent with the idea that an endocytic uptake mechanism may be working for these aggregates; it also suggested that the DNA photocleavage properties of **PdL** should be tested. However, DNA agarose gel experiments at different concentrations (20–100 µM), irradiation times (5–60 min) and incubation times (1–24 h) after activation demonstrated that **PdL** neither interacted with DNA in the dark nor cleaved DNA upon irradiation (Supplementary Fig. 19). Interestingly, after incubating **PdL** nanoparticles with pUC19 plasmid DNA (2 mg ml^−1^) at 37 °C in the dark or after green light irradiation, the **PdL** nanoparticles were found to be stable (Supplementary Fig. 20). Overall, these results suggested that the excellent phototoxicity of **PdL** may result more from increased ROS levels in membrane-rich organelles, such as the lysozome or mitochondria, than from DNA damage in the nucleus. Considering these promising in vitro results, further in vivo testing in mouse tumour models was undertaken.

Human skin melanoma is known to be prone to resisting PDT type II treatment by the combination of a hypoxic tumour microenvironment^35^ and melanin-induced quenching of ROS^36^. Hence, **PdL** was evaluated in vivo using human skin melanoma (A375) tumour xenografts in nude mice. Following intravenous tail injection (100 µl; 420 µM in Dulbecco’s modified Eagle’s medium (DMEM) and 10% foetal bovine serum (FBS); 0.9 mg kg^−1^), the mice showed a constant body weight over 20 d (Fig. 3c) and the important organs remained healthy, as determined by haematoxylin and eosin staining (Supplementary Fig. 21), suggesting low systemic toxicity at this compound dose. In the dark group, **PdL** showed moderate tumour growth inhibition, but green light irradiation (520 nm; 100 mW cm^−2^; 10 min; 60 J cm^−2^) performed 12 h after injection of the self-assembled **PdL** strongly inhibited tumour growth (Fig. 3d). Haematoxylin and eosin staining of the irradiated tumours on day 5 revealed that the tumour tissues were dramatically damaged in the **PdL** + light group, while the other groups did not show any remarkable effect. TUNEL staining also demonstrated a decrease in cancer cells in the irradiated tumour and cell killing via apoptosis (Extended Data Fig. 1b). Overall, these experiments demonstrated not only that **PdL** showed excellent antitumour efficacy in an A375 melanoma mouse model but also that it showed very low cytotoxicity to healthy organs, highlighting the high potential of **PdL** DSDSs for anticancer PDT application.

### Uptake, biodistribution and tumour targeting

**Fig. 4:**
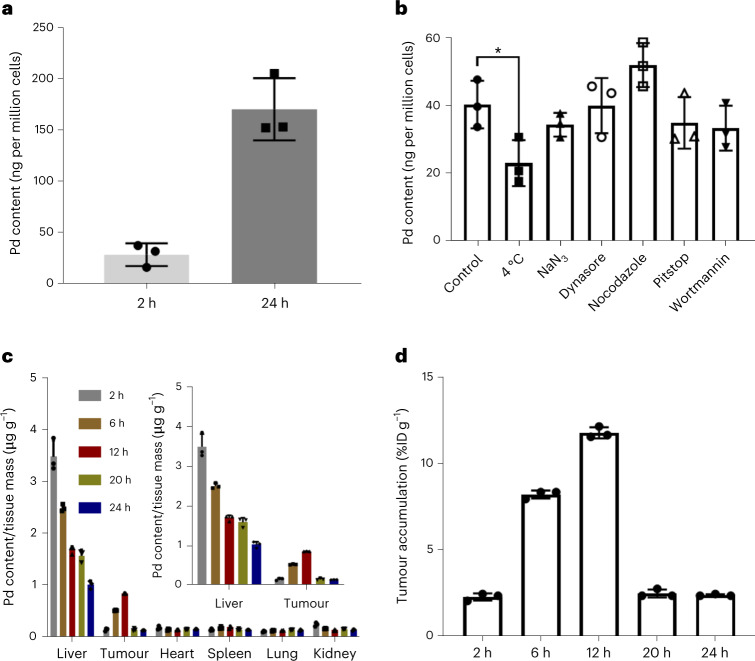
Cellular uptake in vitro; biodistribution, nanoparticle morphology and Pd content; and tumour accumulation of PdL in vivo in mouse A375 tumour xenografts. **a**, Pd content (ICP-MS) of A375 skin melanoma cell monolayers 2 or 24 h after treatment with **PdL** (2 µM). The data represent means ± s.d. of three biological replicates. **b**, Pd content (ICP-MS) of A375 skin melanoma cell monolayers 2 h after treatment with **PdL** (5 µM) in combination with different uptake inhibitors. The data represent means ± s.d. of three biological replicates. Statistical significance was determined by two-way ANOVA (*P < 0.05). **c**, Biodistribution of palladium (inductively coupled plasma optical emission spectroscopy) in different organs of mice at different time points after intravenous tail injection of PdL. The data represent means ± s.d. of three replicates. **d**, Tumour palladium accumulation efficiency in mice at different time points after intravenous tail injection of PdL. The units were determined using the equation %ID g^−1^ = (Pd content of tumour/Pd content of injection solution) × 100%/mass of measured organs. The data represent means ± s.d. of three replicates. In vivo injection conditions: 2.1 µmol kg^−1^, 420 µM, 100 µl DMEM (10% FBS) and 0.9 mg kg^−1^. Source data

The low systemic dark toxicity and high antitumour PDT efficacy of **PdL** stimulated us to check drug uptake in vitro and in vivo using inductively coupled plasma mass spectrometry (ICP-MS). The cellular uptake of **PdL** (2 µM) was found to be time dependent, with the Pd content in A375 cells increasing from 29 ngPd per million cells at 2 h to 172 ngPd per million cells at 24 h (Fig. 4a). It was also temperature dependent, with a decrease to 23 ngPd per million cells at 4 °C 2 h after treatment (5 µM) compared with 41 ngPd per million cells at 37 °C (Fig. 4b). Further coincubation experiments (Fig. 4b) showed that active internalization occurred via clathrin-mediated endocytosis (pitstop) and micropinocytosis (wortmannin). Altogether, these results highlighted that both energy-independent and energy-dependent cellular uptake took place in vitro, suggesting that **PdL** may pass through the cell membrane as both isolated molecules and nanoaggregates.

**Extended Data Fig. 2:**
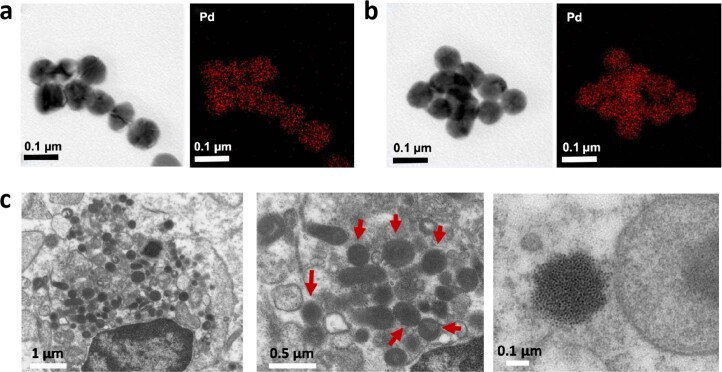
EM images of PdL nanoparticles detected in DMEM, blood and tumor site. (a, b) TEM and EDX element mapping images of nanoparticles detected in DMEM (10% FBS) and in the blood of mice 5 min after treatment with **PdL**. (c) EM images at different magnifications of slices of A375 tumors in mouse xenografts 12 h after intravenous tail injection of **PdL**. Nanoparticles are indicated by red arrows.

An essential question at this stage was to understand whether the nanoparticles formed by **PdL** in cell-growing medium in vitro would also form in a living mouse. Thus, the presence and morphology of nanostructures in the bodies of mice injected with **PdL** were investigated in more detail. First, blood samples taken from the eye socket of mice 5 min after intravenous tail injection of **PdL** in DMEM solution (100 µl; 0.9 mg kg^−1^) showed roughly spherical, high-contrast nanoparticles characterized by an average size of 181 ± 75 nm (Supplementary Fig. 22). Similar to those found in the injected DMEM solution, these nanoparticles were rich in palladium according to energy-dispersive X-ray (EDX) element mapping analysis (Extended Data Fig. 2a), which strongly suggested that they contained **PdL**. As proof of this concept, we also injected the maximum permitted volume (10 µl) of a pure, non-aggregated DMSO solution of **PdL** (4.2 mM) into a mouse to check whether molecules of **PdL** would self-assemble as nanoparticles upon introduction in the blood. Blood collection after 5 min, followed by TEM, EDX and scanning electron microscopy analysis (Extended Data Fig. 2b and Supplementary Fig. 23), demonstrated that palladium-rich round nanoparticles with a diameter of ~100 nm had indeed formed, similar to the nanoparticles observed upon intravenous injection of **PdL** in DMEM. Altogether, these results suggested that molecules of **PdL** spontaneously aggregated into nanoparticles upon injection in the blood, where they remained self-assembled during circulation. The biological half-time of these **PdL** nanoparticles in the blood was found to be ~7.9 h (Supplementary Fig. 26), which is rather long and notably much longer than that found for many small-molecule anticancer drugs (typically below 1 h). At 12 h after tail vein injection of **PdL**, the A375 tumour was sectioned and imaged by electron microscopy. These images (Extended Data Figs. 2c; 1 and 0.5 µm scales; indicated by red arrows) showed dark nanosized spots in the lysozomes of the cancer cells, with an average diameter of 260 ± 75 nm (slightly larger than the diameter of nanoparticles in the blood). These dark spots were not observed in the untreated control group (Supplementary Fig. 24); thus, we interpreted them as palladium-containing nanoparticles. To verify this hypothesis, we performed EDX analysis of these nanoparticles (Supplementary Fig. 25), which confirmed that the cancer cells in the tumour had indeed taken up **PdL** nanoparticles. Overall, the presence of nanoparticles both in the blood and in the tumour tissue of mice treated with **PdL** is proof that the Pd⋯Pd interaction causing the self-assembly of the molecule in medium is strong enough to keep the nanostructures in circulating blood, which leads to delivery of the prodrug to the tumour.

To quantify tumour delivery, the biodistribution of Pd was determined by ICP-MS in A375 mouse xenografts several hours (2, 6, 12, 20 and 24 h) after intravenous tail injection of **PdL** in DMEM. As shown in Fig. 4c, the complex showed low accumulation (below 0.27 µg g^−1^ tissue) in the heart, kidney and lung, while the liver showed significantly higher accumulation (above 1.0 µg per per gram of tissue), as expected considering its role in the detoxification and metabolism of exogenous substances. Noticeably, the accumulation level of **PdL** in the liver gradually decreased over time. Meanwhile, the tumour tissue showed an increasing Pd accumulation from 0.17 µg per gram of tissue after 2 h to a peak of 0.87 µg per gram of tissue at 12 h, which corresponded to an impressive 10.2% of the injected dose per gram (% ID g^−1^) (Fig. 4c,d), and finally decreased to 0.17 µg per gram of tissue at 24 h. These results highlight that the long circulation time in the blood (~8 h) leads to an extraordinary tumour accumulation rate of the **PdL** nanoparticles, which peaks at 12 h. These data suggest that the enhanced permeability and retention effect, conjugated with the high drug loading of the nanoparticle, explains the high tumour accumulation of the compound, as the **PdL** nanoparticles do not contain any active tumour-targeting molecules. In conclusion, **PdL** appears to be a particularly well-performing DSDS characterized by easy synthesis and formulation in biocompatible buffer, high drug-loading efficiency of the self-assembled nanoparticles, low systemic toxicity to the tumour-bearing mouse for these nanoparticles and excellent tumour accumulation and antitumour efficacy upon light irradiation using a drug-to-light interval of 12 h.

## Conclusion

One main advantage of DSDSs is their high drug-loading capacity, as the nanoparticles are mostly composed of drug molecules. However, with traditional DSDSs, such high drug-loading capacity may come with high toxicity to blood-filtering organs such as the liver or kidneys^37^, as each nanoparticle brings into a cell many toxic molecules. Light-activated DSDSs bring a solution to this problem, as the self-assembling drug only becomes toxic after light irradiation^38^. As the liver remains in the dark, high hepatic uptake is not a problem for **PdL**, provided liver clearance occurs, which is suggested in Fig. 4c. Light activation hence provides a dramatic advantage compared with traditional DSDSs.

Light-activated DSDSs can also be analysed from the point of view of PDT. Of course, PDT treatment offers a patient-friendly alternative to chemotherapy, with the potential to inhibit tumour proliferation while minimizing side effects by selective light irradiation of tumour tissue^39^. However, photosensitizer molecules taken up in healthy tissues also lead to undesired photosensitivity (for example, of the skin) for the patient—a typical side effect of PDT with Photofrin^40^. It is hence essential that the photosensitizer be delivered with high efficacy to the tumour tissue. Many reports have demonstrated that nanoconjugates enable an increase in the tumour accumulation of molecular drugs, including PDT photosensitizers^41,42^. However, the low average drug-loading capacity (typically 20 wt%) and tumour accumulation efficacy (a median of 0.7% ID) achieved by classical drug delivery nanosystems^20^ suggested the urgency to develop new drug delivery nanoconjugates, also in the context of PDT.

The drug delivery properties of **PdL** include a long circulation time (>12 h) with good levels of accumulation in the tumour tissue (up to 10.2% ID g^−1^). Photochemically speaking, unlike porphyrins, the **PdL** complex maintains excellent photodynamic properties in the aggregated state, even in a hypoxic tumour microenvironment. With these results in hand, we conclude that the metallophilic interaction can potentially be used to build high-performance supramolecular nanocarriers with improved tumour accumulation and that the Pd⋯Pd interaction observed with complexes such as **PdL** can generate photodynamic systems that conserve their phototoxic properties under hypoxia.

## Methods

### Ethics declarations

This study was conducted following the *Guide for the Care and Use of Laboratory Animals*^43^. All animal experiments were performed under guidelines approved by the ethics committee of the Dalian University of Technology. The maximal tumour size permitted was 2,000 mm^3^ (and the maximum burden was 10% of the body weight), but this limit was never reached in the experiments described below; the maximum tumour size in the experiments described below was ~1,000 mm^3^.

### General materials

All reagents were purchased from commercial vendors. The reactants and solvents were used without further purification. All ^1^H NMR and ^13^C attached proton test NMR results were obtained on Bruker DPX 300 spectrometers. Chemical shifts are indicated in ppm relative to the residual solvent peak. Electrospray ionization mass spectra (ESI-MS) were recorded using an MSQ Plus spectrometer in positive ionization mode. The TEM experiments were carried using a JEOL 1010 (10 kV) transmission electron microscope with a Formvar/carbon-coated copper grid from Polysciences. Ultraviolet–visible spectra were recorded on a Cary 50 spectrometer from Varian. The emission spectra were measured using an FLS900 spectrometer from Edinburgh Instruments. The singlet oxygen emission spectra were measured on a special custom-built setup that was described previously^31^. The spectra data were analysed using Origin 8. The human cancer cell lines A549 (lung carcinoma), A431 (skin carcinoma) and A375 (malignant melanoma) were distributed by the European Collection of Authenticated Cell Cultures and purchased from Sigma–Aldrich. DMEM (with and without phenol red and without glutamine), glutamine-S (200 mm), tris(hydroxymethyl)aminomethane (Tris base), trichloroacetic acid, glacial acetic acid and sulforhodamine B were purchased from Sigma–Aldrich. Opti-MEM Reduced Serum Medium without phenol red was obtained from Gibco. The measurements of complexes on photocytotoxicity were performed according to the literature^31^. The annexin V/propidium iodide double staining assay was purchased from Bio-Connect. The FractionPREP Cell Fractionation Kit was obtained from BioVision Incorporated. The cytotoxicity data were analysed with GraphPad 8. The confocal microscopy and electron microscopy images were analysed using ImageJ 1.52v.

### Synthesis of H_2_L

A mixture of 2-(3-bromophenyl)pyridine (329 mg; 1.41 mmol), Pd(dba)_2_ (81 mg; 0.14 mmol), racemic BINAP (106 mg; 0.17 mmol) and KO*t*-Bu (1,574 mg; 14 mmol) was partially dissolved in dry toluene (28 ml) under an N_2_ atmosphere. The mixture was stirred for 10 min, then 3-(2-pyridyl)aniline (230 mg; 1.35 mmol) was added, followed by heating the reaction mixture to 95 °C. After 3 d of stirring, the brown mixture was cooled down. Demineralized water (75.0 ml) was added and the mixture was stirred for 1 h. The H_2_O layer was separated from the toluene layer. The H_2_O layer was extracted with EtOAc (100 ml) three times and the toluene and EtOAc layers were combined, followed by rotary evaporation of the solvents. The crude product was purified by silica chromatography using pentane–EtOAc mixtures (2:1; *R*_f_ = 0.3) as the eluent, to afford 290 mg of the target compound **H**_**2**_**L**^**1**^ (yield: 0.90 mmol; 67%). ESI-MS (cation): *m/z* calculated = 324.2 (C_22_H_17_N_3_ + H^+^); found = 324.7. ^1^H NMR (300 MHz; DMSO-*d*_6_): *δ* = 8.65 (dt, *J* = 4.7, 1.4 Hz, 2H), 8.49 (s, 1H), 7.97–7.81 (m, 6H), 7.53 (dt, *J* = 7.8, 1.3 Hz, 2H), 7.42–7.30 (m, 4H), 7.20 (dd, *J* = 7.8, 2.3 Hz, 2H). ^13^C attached proton test NMR (75 MHz; DMSO-*d*_6_): *δ* = 156.1, 149.5, 143.8, 139.7, 129.6, 122.6, 120.2, 120.2, 118.1, 117.4, 115.1.

### Synthesis of PdL

A mixture of **H**_**2**_**L** (90 mg; 0.28 mmol) and Pd(OAc)_2_ (63 mg; 0.28 mmol) in glacial acetic acid was refluxed for 24 h at 135 °C under an N_2_ atmosphere to give a yellowish green solution. Then, the solvent was rotary evaporated. The crude product obtained was purified by silica chromatography using dichloromethane/MeOH mixtures (vol/vol = 100:1.5; *R*_f_ = 0.3) as the eluent to afford 67 mg of the target complex **PdL** (yield: 0.15 mmol; 56%). ESI-MS (cation): *m/z* calculated = 428.0379 (C_22_H_15_N_3_Pd + H^+^); found = 428.0374. ^1^H NMR (300 MHz; DMSO-*d*_6_) *δ* = 9.20 (s, 1H), 8.94 (d, *J* = 5.4 Hz, 2H), 8.20 (d, *J* = 8.1 Hz, 2H), 8.10–8.00 (m, 2H), 7.49 (ddd, *J* = 7.1, 5.5, 1.3 Hz, 2H), 7.46–7.37 (m, 2H), 7.14 (t, *J* = 7.6 Hz, 2H), 7.00 (dd, *J* = 7.9, 1.1 Hz, 2H). ^13^C NMR (75 MHz; DMSO-*d*_6_) *δ* = 163.77, 148.75, 146.55, 139.56, 138.71, 137.87, 124.64, 122.74, 119.50, 115.14, 114.52. Elemental analysis calculated for **PdL**: C = 61.77, H = 3.53, N = 9.82; found: C = 61.93, H = 3.64, N = 9.60.

### Computational details

The DFT and TDDFT calculations were carried out using the Amsterdam Density Functional software (ADF 2019, SCM, Theoretical Chemistry, Vrije Universiteit; http://www.scm.com), the hybrid PBE0 functional, a triple zeta basis set with polarization (TZP) for all atoms, COSMO to simulate the solvent effect (in methanol) and ZORA to account for scalar relativistic effects. Moreover, density-dependent dispersion correction was included for the geometry optimization. The *x,* *y* and *z* values of the minimized coordinates of the monomer and dimer of **PdL** are provided in Supplementary Tables 3 and 4 and analysis of the TDDFT results is provided in Supplementary Table 5. To calculate the energy of the [**H**_**2**_**L**]_2_ dimer, the two palladium atoms in the minimized geometry of the [**PdL**]_2_ dimer were replaced with four hydrogen atoms and, without further optimization, a single-point energy calculation was performed. The dimerization energies (*E*_dimer_ − 2*E*_monomer_) of both dimers at the same level of theory were then compared.

### Cryo-EM measurement

We applied 6 µl of the sample ([**PdL**] = 25 µM) to a freshly glow-discharged carbon 200 mesh Cu grid (Lacey Carbon Film; Electron Microscopy Sciences, Aurion). Grids were blotted after a 10-s wait for 3 s at 99% humidity in a Vitrobot plunge freezer (FEI Vitrobot Mark III; Thermo Fisher Scientific). Cryo-EM images were collected on a Titan Krios operating at 300 kV at a nominal magnification of 33,000× or 81,000×, yielding a pixel size at the specimen of 3.5 or 1.4 Å, respectively (The Netherlands Centre for Electron Nanoscopy, Leiden University).

### In vivo tumour inhibition experiments

Female 3-week-old BALB/c mice were originally purchased from Vital River Laboratory Animal Center (Beijing, China). The mice were kept under specific pathogen-free conditions with free access to standard food and water for 2 weeks at 20–21 °C, under 40–60% relative humidity and with a 12 h dark/12 h light cycle. The mice were used for experiments when their weight reached ~20 g. This study was conducted following the *Guide for the Care and Use of Laboratory Animals*^43^. All protocols for the animal studies conformed to the *Guide for the Care and Use of Laboratory Animals*^43^. All animal experiments were performed under guidelines approved by the ethics committee of the Dalian University of Technology. The tumour model was established by inoculating 5 × 10^7^ A375 melanoma cells suspended in 100 μl phosphate-buffered saline (PBS) at the right flank region of each mouse, to obtain mouse A375 melanoma implants. After 3 weeks, the tumour volumes were ~100 mm^3^_._ The tumour volume (*V*) was calculated using formula *V* = *L*/2 × *W*^2^ after measuring the tumour length (*L*) and width (*W*)^3^. The mice were then randomly divided into four groups (vehicle control, 520 nm light, **PdL** and **PdL** + 520 nm light, with four mice in each group). The injectable **PdL** solution was prepared by diluting the **PdL** stock DMSO solution (4.2 µM) to 420 µM using DMEM medium containing 10% vol/vol FBS and 1% vol/vol penicillin/streptomycin. The mice were treated through tail intravenous injection with DMEM (vehicle control and 520 nm light groups) or **PdL** (2.1 µmol kg^−1^; 420 µM; 100 µl DMEM medium (10% FBS); 0.9 mg kg^−1^) (**PdL** dark and **PdL** + 520 nm light groups). After 12 h injection, 520 nm irradiation (100 mW cm^−2^; 5 min) was then carried out twice, with an interval of 5 min, for the light groups. Thus, the total light dose for each treatment was 100 mW cm^−2^ for 10 min at 60 J cm^−2^. These treatment and irradiation steps were replicated at days 0, 7 and 14, respectively. On day 5, one mouse in each group was killed and the tumour was taken up and fixed with paraformaldehyde (10% vol/vol), then sectioned into slices and analysed via haematoxylin and eosin or TUNEL protocols to evaluate the tumour cell damage and apoptosis conditions. The tumour volume and body weight of the remaining mice (*n* = 3) were measured and recorded and the average tumour volume and body weight were calculated over 20 d. Finally, these mice were also killed and the healthy organs were removed, fixed with paraformaldehyde (10% vol/vol) and then sectioned into slices and analysed via the haematoxylin and eosin protocol to determine the side effects of treatment.

### Mouse blood electron microscopy imaging experiments

Tumour-bearing mice were treated with **PdL** cell medium solution (2.1 µmol kg^−1^; 420 µM; 100 µl DMEM medium (10% FBS); 0.9 mg kg^−1^) or pure DMSO solution (2.1 µmol kg^−1^; 4.2 mM; 10 µl; 0.9 mg kg^−1^) through intravenous tail injection. After 5 min, 1 ml blood was taken up from the eye socket and diluted to 5 ml with PBS. After centrifugation (1,500 r.p.m. (239*g*) for 10 min), the supernatant was collected and the left part was washed with PBS (5 ml) and centrifuged (1,500 r.p.m. (239*g*) for 10 min) again twice more to obtain the supernatant PBS solution. These PBS solutions were then combined and centrifuged at a speed of 10,000 r.p.m. (10,621*g*) for 10 min. After removing the supernatant, 200 µl PBS was added and mixed well. Then, the solutions were transferred to the TEM grids. For the preparation of TEM samples, a drop (15 µl) of the solution was added to the grids (formvar/carbon 200 mesh; copper) and kept for 2 min, then the excess liquid on the grid was removed using filter paper and dried for 2 h for TEM measurement. The TEM measurements were carried out under vacuum conditions (Hitachi H-7650).

### Mouse tumour electron microscopy imaging experiments

One tumour-bearing mouse was treated with **PdL** (2.1 µmol kg^−1^; 420 µM; 100 µl DMEM medium (10% FBS); 0.9 mg kg^−1^) through intravenous tail injection. After 12 h, the mouse was sacrificed, the tumour tissue was collected and then fixed with a biological TEM fixation solution (Wuhan Servicebio). Next, the tumour tissue was split into small pieces with a volume of ~1 mm^3^ and fixed again using 1% osmic acid phosphate buffer solution for 2 h, followed by dehydration with ethanol (vol/vol = 30, 50, 70, 80, 95 or 100%; 20 min per group) and acetone twice (15 min). The prepared samples were then treated with acetone/epon-812 embedding medium at a ratio of 1:1 for 2 h and 1:2 for 12 h, and then pure epon-812 solution for another 5 h at 37 °C. Next, the tissue-containing embedding medium was filled in the embedding mould for 24 h at 37 °C and another 48 h at 60 °C. The obtained tissue-containing resin was then sectioned into slices with a thickness of ~60–80 nm via ultramicrotome (Leica EM UC7) and moved to the copper grid (150 mesh). The obtained grids were stained with 2% uranyl acetate ethanol solution for 8 min and 2.6% lead citrate solution for another 8 min. Next, the grids were dried at room temperature and observed using a JEOL JEM-2100 transmission electron microscope (Japan).

### Reporting summary

Further information on research design is available in the Nature Portfolio Reporting Summary linked to this article.

## Online content

Any methods, additional references, Nature Portfolio reporting summaries, source data, extended data, supplementary information, acknowledgements, peer review information; details of author contributions and competing interests; and statements of data and code availability are available at 10.1038/s41557-023-01199-w.

## Supplementary information

Supplementary Information Description of the supplementary experiments, including Supplementary Figs. 1–26 and Tables 1–8.

Reporting Summary

Supplementary Data 1 CIF file for the X-ray structure of PdL.

Supplementary Data 2 CheckCIF file for the X-ray structure of PdL.

Supplementary Fig. 27 Unprocessed protein gel for Supplementary Fig. 10.

Supplementary Fig. 28 Unprocessed DNA gel for Supplementary Fig. 19.

## Data Availability

All of the relevant data gathered during this study have been deposited in the Zenodo repository (https://zenodo.org/record/7032728) or can be obtained from the corresponding authors upon request. Crystallographic data for the structure reported in this Article have been deposited at the Cambridge Crystallographic Data Centre under deposition number CCDC 2142714 (**PdL**). Source data are provided with this paper.

## Source data

Source Data Fig. 1 Statistical source data for TDDFT calculation and absorption, emission and self-assembly spectra in Fig. 1d–g.
Source Data Fig. 2 Statistical source data for DLS, 24-h absorption, stability in cell medium and PDT mechanism determination spectra in Fig. 2a–c,f–h.
Source Data Fig. 3 Statistical source data for anticancer cytotoxicity of PdL in 2D monolayers and 3D tumour spheroids, apoptosis, mouse body weight and mouse tumour growth curves in Fig. 3a–d.
Source Data Fig. 4 Statistical source data for cell uptake, uptake inhibition, mouse body distribution and tumour accumulation percentage in Fig. 4a–d.
