# Supplementary material for: In vivo metallophilic self-assembly of a light-activated anticancer drug
Source: Nat Chem. 2023 May 11;15(7):980–7. doi: 10.1038/s41557-023-01199-w (PMC10322715; doi:10.1038/s41557-023-01199-w)
Supplement: Supplementary file 1 — Description of the supplementary experiments, including Supplementary Figs. 1–26 and Tables 1–8. [file 41557_2023_1199_MOESM1_ESM.pdf]

nature chemistry

Article

https://doi.org/10.1038/s41557-023-01199-w

In vivo metallophilic self-assembly of a light-activated anticancer drug

In the format provided by the authors and unedited

Contents of the Supplementary Information

1. Experiments 1
2. NMR characterization of ligands and metal complexes 8
3. Single crystal X-ray crystallography 10
4. DFT and TDDFT calculation of PdL 12
5. Photophysical properties of PdL 15
6. Self-assembly of PdL in solvent mixtures 17
7. Characterization of PdL in Opti-MEM complete medium 19
8. Photocytotoxicity of PdL and cell death mechanism determination in cancer cells 21
9. Tumor xenografts experiments 28
10. References 31

1. Experiments

Transient absorption. The femtosecond pump-probe transient absorption (TA) measurements were performed using a regenerative amplified Ti:sapphire laser system (Coherent; 800 nm, 70 fs, 6 mJ/pulse, and 1 kHz repetition rate) as the laser source and a femto-100 spectrometer (Time-Tech LLC). Briefly, the 800 nm output pulse from the regenerative amplifier was split into two parts. One part was second harmonic generation (SHG) by a BBO crystal to produce 400 nm laser, which is the excitation laser in our experiments. Another part with less than 10% was attenuated with a neutral density filter and focused into a crystal to generate a white light continuum (WLC) used for probe beam. The probe beam was focused with an Al parabolic reflector onto the sample. After the sample, the probe beam was collimated and then focused into a fiber-coupled spectrometer with CMOS sensors and detected at a frequency of 1 kHz. The intensity of the pump pulse used in the experiment was controlled by a variable neutral-density filter wheel. The delay between the pump and probe pulses was controlled by a motorized delay stage. The pump pulses were chopped by a synchronized chopper at 500 Hz and the absorbance change was calculated with two adjacent probe pulses (pump-blocked and pump-unblocked). The linear polarization angle difference between the pump and probe light keeps magic angle (54.7°) to record the isotropic response.

Nanosecond TA was performed with the EOS spectrometer (Ultrafast Systems LLC). The pump beam is generated in the same way as the femtosecond TA experiment described above. A different white light continuum (380-1700 nm, 0.5 ns pulse width, 2 kHz repetition rate) was used, which was generated by focusing a Nd:YAG laser into a photonic crystal fiber. The delay time between the pump and probe beam was controlled by a digital delay generator (CNT-90, Pendulum Instruments).

Time-resolved photoluminescence. Time-resolved photoluminescence (PL) decay curves within 1 μs scale were measured by a home-made time-correlated single photon counting (TCSPC) set-up. The excitation source is a supercontinuum laser (100 ps pulse width, 1 MHz repetition rate) with proper optical filters. The PL photons were collected by a lens, filtered and then detected by an avalanche photodiode detector (APD).

Time-resolved PL decay curves within 800 μs scale were measured by a static/transient fluorescence spectrometer (Edinburgh instruments; FLS 1000-stm). The excitation source is a pulse Xe lamp (2 μs, 40 Hz) with a monochromator. Along the axis perpendicular to the excitation light, the PL was collected by a lens, separated by a monochromator and finally detected by a PMT.

Kinetic Fitting of Transient Absorption Data. Under the degassed condition, the triplet main decay process is phosphorescence. Thereby, the rate equation can be depicted as eq. (S1),

$$\frac{d[T]}{dt} = -k_p[T] \qquad \text{(S1)}$$

Where the [T] is the triplet concentration, and k_p is the phosphorescence radiation rate ($\frac{1}{k_p}$ is the phosphorescence lifetime). The solution of eq. (S1) is eq. (S2).

$$T(t) = e^{-k_p t} \qquad \text{(S2)}$$

Based on this, the decay curves for TA and PL under the degassed condition were fitted by 1-exponential function to obtain the decay lifetime and rate. Under the air condition, the triplet decay path processes are phosphorescence and charge transfer, the rate equation can be depicted as eq. (S3),

$$\frac{d[T]}{dt} = -(k_p + k_{CT})[T] \qquad \text{(S3)}$$

Where k_{CT} is the charge transfer rate, the solution of eq. (S3) is eq. (S4),

$$T(t) = e^{-(k_p + k_{CT})t} \qquad \text{(S4)}$$

Therefore, the PL decay curves under the air condition were fitted by mono-exponential function to obtain the charge transfer rate. However, TA decay curve under the air condition

contains the triplet decay and the cation decay because of the PIA feature overlap between triplet and cation. Thereby, the TA decay curve can be illuminated by eq. (S5),

$$TA(t) = A_1 e^{-(k_p + k_{CT})t} + A_2 e^{-k_{CR} t}$$

Where the A_1 and A_2 are the contributions of triplet and cation towards PIA, respectively, k_{CR} is the charge recombination rate of the cation. Therefore, the TA decay curves under the air condition were fitted by 2-exponential function to obtain the charge recombination rate.

Nanoparticle protein content determination. The protein content of **PdL** nanoparticle was determined by protein gel and commercial pierce BCA protein assay kit according to literature.[1] For the protein gel experiment, 100 μL of PdL solution (4.2 mM, DMSO) was mixed with 900 μL of DMEM medium (containing 10% v/v FBS). Then the solution was centrifuged for 30 min (8000 rpm, 7104 g, 4 °C) and washed with 1 mL of PBS. This step was repeated three times. To collect the corona proteins, the nanoparticles were then incubated at 200 μL of 62.5 mM Tris-HCl buffer containing 2% sodium dodecyl sulfate for 5 min at 95°C. Then the solution was centrifuged for 30 min (8000 rpm, 7104 g, 4 °C) to collect the bare **PdL** nanoparticle in precipitate and isolated corona proteins in supernatant, separately. Then the protein gel was performed to evaluate the protein content in the washed nanoparticle and supernatant solution.

For the pierce BCA protein assay kit, 20 μL of **PdL** solution (4.2 mM, DMSO) was mixed with 180 μL of DMEM medium (containing 10% v/v FBS) and incubated at 37 °C for 1 h, 2 h and 4 h. Then the same method as above was used to collect the wash nanoparticle and supernatant corona protein solution. After that, the Pierce BCA protein assay kit was used according to the protocol of manufacturer to determine the total protein content in each group. Results are shown in Supplementary Figure 10.

2D-monolayer photocytotoxicity experiments. For the cytotoxicity assay, Opti-MEM complete medium without phenol red was used, supplemented with 2.5% v/v fetal calf serum (FCS), 0.2% v/v penicillin/streptomycin (P/S), and 1% v/v Glutamine). Briefly, 100 μL Opti-MEM complete medium suspensions of A549 (5000 cells), A431 (8000 cells), or A375 (5000 cells) cells, were seeded into 96-wells plates and separated as dark or light groups, and incubated in the normoxic (21% O_2, 37 °C) or hypoxic (1% O_2, 37 °C) incubators. After 24 h, the cells were treated with **PdL** (100 μL) in a series of concentrations. At 48 h, the cell plates in the light group were irradiated with 520 nm green light with a dose of 13 J/cm^2 (normoxic-2D: 20 min, 10.92 mW/cm^2; hypoxic-2D: 32 min, 6.90 mW/cm^2), in normoxic (21% O_2) or hypoxic (1% O_2) conditions, while the dark group was kept in the dark. After irradiation, the

cells were incubated in the dark for another 48 h. Then 100 μL of TCA fixation solutions (10% w/v) were added to the wells, and the plates were kept at 4 °C for 24 h. The photocytotoxicity of the complex was determined *via* the sulforhodamine (SRB) assay, and the normoxic or hypoxic half-maximal effective concentrations EC_{50} were obtained *via* Graphpad 8 using the dose-response two-parameter Hill-slope equation $Y = 100/(1 + 10^{(log_{10}EC_{50}-X) \times Hill\ Slope})$. Data are averages (n=3) with 95% confidence intervals (in μM) over three independent experiments.

3D tumor spheroids viability assay. 100 μL Opti-MEM complete medium suspensions of A549 (500 cells), A431 (500 cells), or A375 (300 cells in normoxic conditions, 1000 cells in hypoxic conditions) cells were seeded into 96-well round-bottom Corning spheroid microplates and split as dark or light groups. Each plate was incubated for 3 days in normoxic or hypoxic conditions, to obtain 3D tumor spheroids. Then, the spheroids were treated with **PdL** (100 μL Opti-MEM complete medium) in a concentration series (0, 0.05, 0.25, 0.5, 1, 1.25, 2.5, 5, 12.5, 25). 24 h later, the plates of the light group were irradiated with 520 nm green light with a dose of 13 J/cm^2 (normoxia-3D spheroid condition: 32 min, 6.90 mW/cm^2; hypoxia-3D spheroid condition: 55 min, 3.99 mW/cm^2) and incubated for another 48 h. Then a CellTiter Glo 3D solution (50 μL/well) was added to each well to stain the 3D tumor spheroids. After 30 min shake on an IKA Vibrax shake at 500 rpm at room temperature, the luminescence in each well was measured by a Tecan Microplate Reader. Half-maximal effective concentrations (EC_{50}) for 3D tumor spheroids growth inhibition were calculated by fitting the CellTiter Glo3D dose-response curves using the same non-linear regression function as in 2D (two-parameter Hill-slope equation) as implemented in Graphpad Prism 8. Data are averages (n=3) with 95% confidence intervals (in μM) over three independent experiments.

Mode of cell death study using flow cytometry. 2×10^5 A375 cells were seeded in 12-well plates that were separated into dark and light groups, and incubated in normoxic condition. After 24 h, the cells were treated with **PdL** at a final concentration at 0.5 μM or 2 μM, and incubated for 24 h. Then, the light groups were irradiated with 520 nm green light with a dose of 13 J/cm^2 (20 min, 10.92 mW/cm^2), and incubated in normoxic condition for 2 h, 4 h or 24 h. Afterward, the cells in all groups were harvested with trypsin and stained with Annexin V/propidium iodide dyes for 15 min. The apoptosis status of cells was then determined via flow cytometry immediately. Parameter "GRN-B" (488 nm excitation, 525±30 nm emission) "RED-B" (488 nm excitation, 661±15 nm emission) were used for fluorescence measurements to match with the known excitation/emission wavelengths of Annexin V-FITC

(494/518 nm) and propidium iodide (535/617 nm). All flow cytometry data were processed using FlowJo10.

Cellular uptake experiments. To measure simple cellular uptake, A375 cells (2×10^5) were seeded in 12-well plates and incubated for 48 h. Then the cells were treated with **PdL** (2 μM, 1 mL) for 2 h or 24 h. After that, the cells were washed by PBS for one time, and then harvested and centrifuged. After removing the supernatant, 0.5 mL of 65% HNO_3 was added to lyse the cell pellets with an overnight shake. For cellular uptake inhibition experiments, the cells were pretreated with different inhibitors for 1 h (NaN_3 (1 mg/mL), pitstop 2 (20 μM), dynasore (80 μM), nocodazole (40 μM), and wortmannin (4 μM)), or incubated at 4 °C for 30 min. Then, the cells were treated with **PdL** (5 μM) and incubated either in normoxic conditions (37 °C, 5% CO_2, 21% O_2, 100% humidity) or at 4 °C (in the air condition) for another 2 h. After that, the cells were harvested, centrifuged and lysed using the same method as in absence of inhibitor. Then, 9.5 mL of milli-Q water was added to the cell lysis solution to lower the HNO_3 concentration to 3.25% (v/v). The Pd content in the solution samples was measured *via* ICP-MS (NexION 2000, PerkinElmer).

Intracellular ROS detection. A375 cells (approximately 2×10^5 cells/mL) were seeded into 35 mm diameter confocal dishes. The cells in the dish were incubated with DMEM containing 20 μM **PdL** for 12 h, and then co-cultured with 1 μM DCFH-DA for another 30 min. After that, cells were irradiated by green light (520 nm, 13 J/cm^2, 20 min). Finally, cells were imaged using a confocal laser scanning microscope with a DCF excitation wavelength of 488 nm and an observation window of 500 nm to 600 nm.

Intracellular superoxide anion free radical detection. A375 cells (approximately 2×10^5 cells/mL) were seeded into 35 mm diameter confocal dishes. The cells in the dish were incubated with DMEM containing 20 μM **PdL** for 12 h, and then co-cultured with 10 μM DHE for another 30 min. After that, cells were irradiated by green light (520 nm, 13 J/cm^2, 20 min). Cells were imaged using a confocal laser scanning microscope with a DHE excitation wavelength of 488 nm and an observation window of570 nm to 630 nm.

Intracellular GSH level determination. The PdL-induced GSH concentration variation was determined by using a GSH and GSSG Assay Kit. The A375 cells (1×10^7) in a 13 cm culture dish were treated by PdL at a dose of 20 μM or 10 μM in the dark or under light irradiation (520 nm, 10.9 mW/cm^2, 20 min). The incubation time was set at 12 h and drugs-free cells were set as the control. Then the intracellular GSH concentrations of five groups (20 μM (PdL-Dark and PdL-Light), 10 μM (PdL-Dark and PdL-Light) and Control) were measured according to the manufacturer's suggested protocol.

Cell fractionation and localization of palladium. The experiment was carried out according to the method described in the literature.[2] Briefly, A375 cells (approximately 1×10^7 cells/mL) were seeded into 35 mm diameter confocal dishes. The cells in the dish were incubated with DMEM containing 5 μM PdL for 24 h. After that, the cell fraction (cytosol, membrane, nucleus, and cytoskeletal) was collected using the fractionPREP cell fractionation kit from BioVision according to the instructions of the supplier. Then the fraction samples were treated with 0.5 mL of 65% HNO_3 and shaken at room temperature for 24 h. Then 0.5 mL 30% H_2O_2 was added and heated at 90 °C in an oven for 24 h to dissolve all biological materials. Then the Pd content in each fraction was measured using ICP-MS.

DNA cleavage study by gel electrophoresis. The experiment was done according to previous work.[2] The pUC19 plasmid (2686 bp) used here exists in three forms: supercoiled (SC), single-nicked open circular (OC), and linear dimer (LD). Chloride-free phosphate buffer (PB) was used here to mimic a pseudo intracellular environment. All aliquots were prepared with a final volume of 20 μL and prior to loading 4 μL of 6X loading dye was added. The GeneRuler DNA Ladder Mix molecular weight (MW) marker was prepared by adding 4 μL (2 μg) of the DNA MW marker, 16 μL PB, and 4 μL 6X loading dye. Then, 2 μL of pUC19 plasmid DNA was incubated with 0, 20, 40, or 100 μM PdL, under dark or green light irradiation (520 nm, 10.9 mW/cm^2) for 5, 15, 45, 60 min, respectively. After that, the solutions were directly loaded for DNA gel or incubated in dark for 1, 4, 8, or 24 h at 37 °C. In each well, 10 μL of each sample was loaded in a 1% agarose gel. For each gel, the electrophoresis chamber was filled with 50 mL TBA 5X and 200 mL deionized H_2O. Each gel was run at a constant voltage of 105 V for 90 min. All gels were stained using 20 μL SYBR™ Safe DNA Gel Stain in 200 mL TBA 1X for 30 min with slight shaking. Immediately following staining, the gel was imaged using a BioRad ChemiDoc imaging system (SYBR™ Safe setting). Image Lab software was used to process the images.

Blood circulation study. 100 μL of PdL DMEM solution (420 μM, 10% v/v FBS) was injected into mice via intravenous tail injection. Then mice blood was taken up from the eye socket at 5, 15, 30 min, and 1, 2, 5, 10, 24, 48, 72 h (n=3 for each group) and the weight of blood was measured. The blood samples were lysed in a mixture solution of 65 % HNO_3 (5 mL) and 30 % H_2O_2 (2 mL) at 100 °C. Afterward, each sample was redissolved in 5 mL of water and filtered by 200 μm membrane filter. The Pd content was then determined via ICP-MS, and the blood circulation half-time was calculated according to the equation reported in literature.[4]

Pd distribution determination on mice organs. The mice were treated with **PdL** (2.1 μmol/kg, 420 μM, 100 μL DMEM medium (10% FBS), 0.9 mg/kg) through intravenous tail injection. Then, the mice were sacrificed at 2 h, 6 h, 12 h, or 20 h, or 24 h, and their heart, liver, spleen, kidney, lung, and tumor, were taken. Then, around 1 g of each organ was lysed overnight in a mixture solution of 65 % HNO_3 (5 mL) and 30 % H_2O_2 (2 mL) at 100 °C. Afterward, each sample was evaporated and another 5 mL HNO_3 solution (2 %) was added. The Pd content in each organ or tumor was detected *via* ICP-OES (JY-Horiba ICP-OES Ultima 2).

2. NMR characterization of ligands and metal complexes.

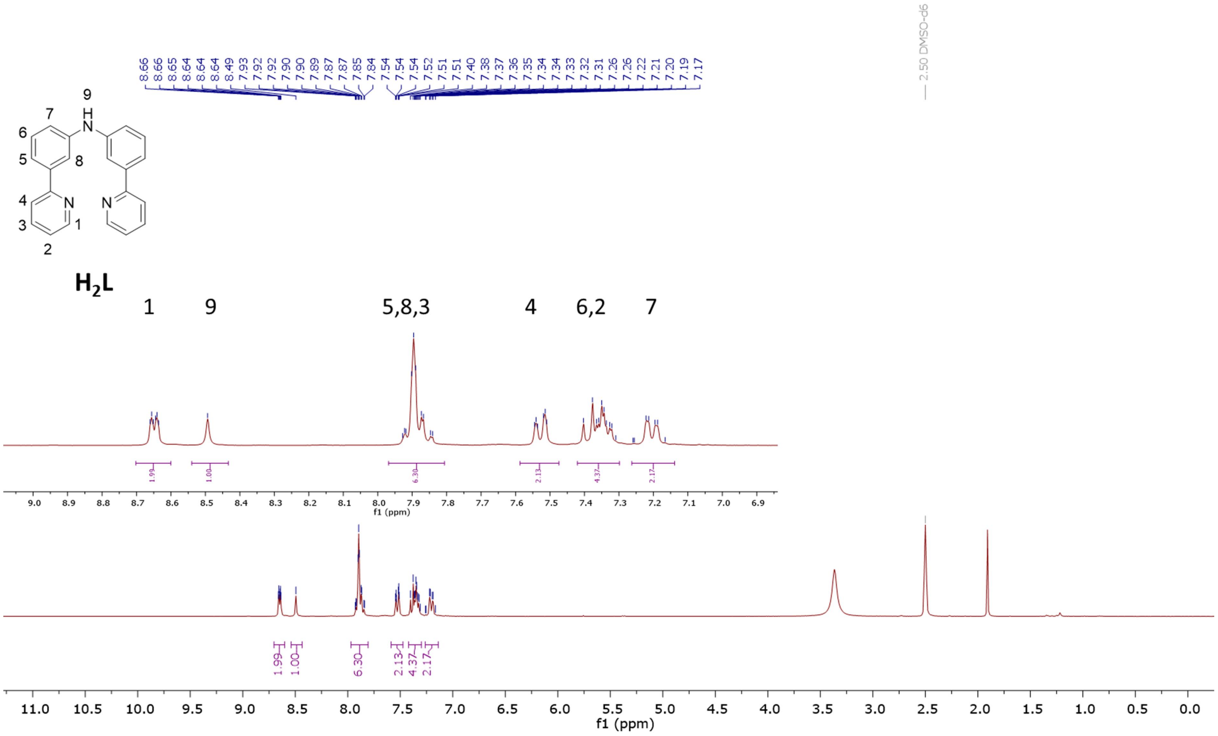

Supplementary Figure 1. ^{1}H NMR of ligand $\mathbf{H_2L}$ in DMSO-d_6.

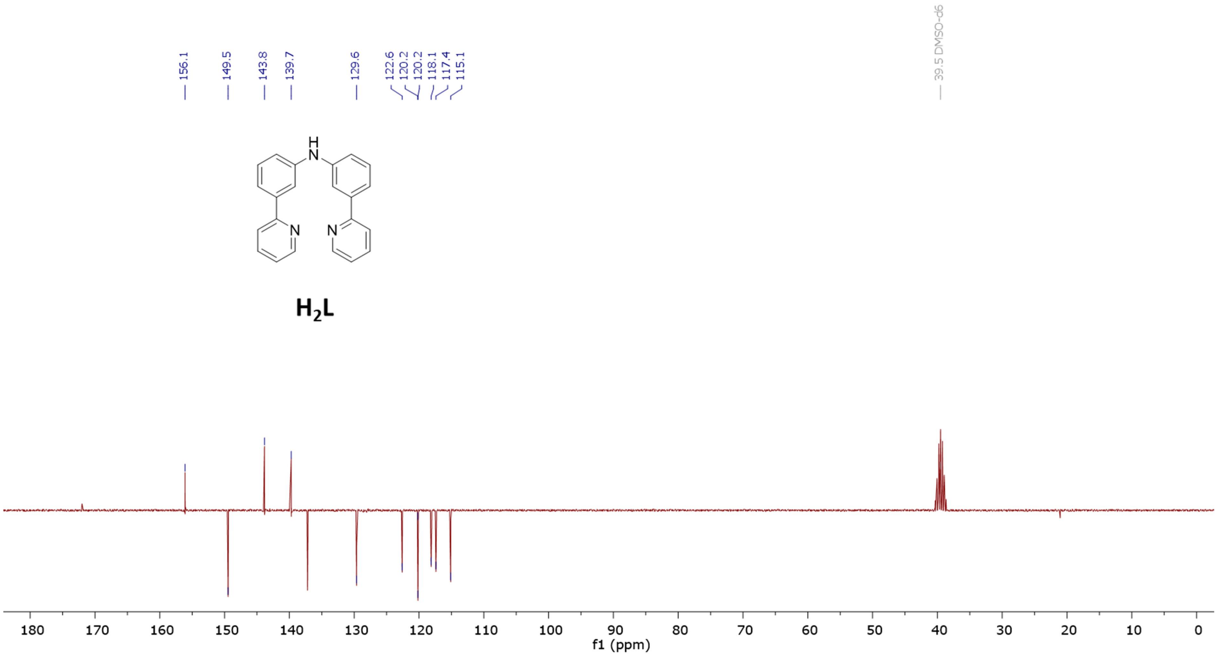

Supplementary Figure 2. ^{13}C-APT NMR of ligand $\mathbf{H_2L}$ in DMSO-d_6.

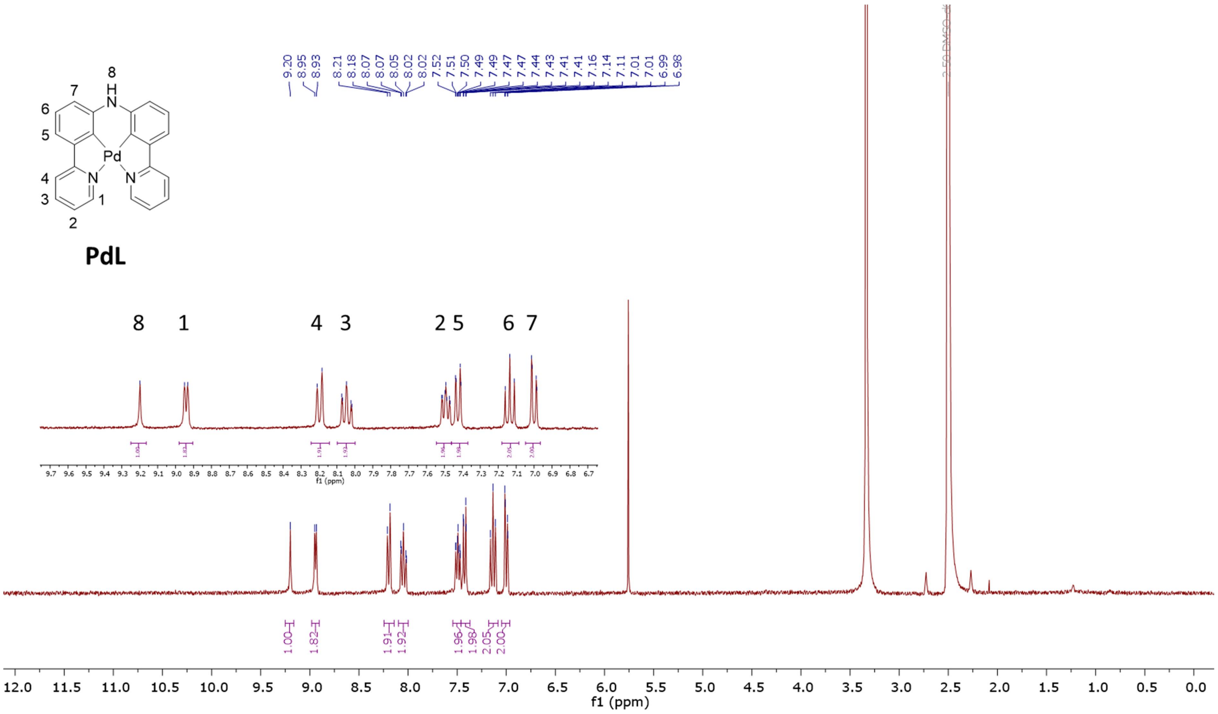

Supplementary Figure 3. ^{1}H NMR of **PdL** in DMSO-d_6.

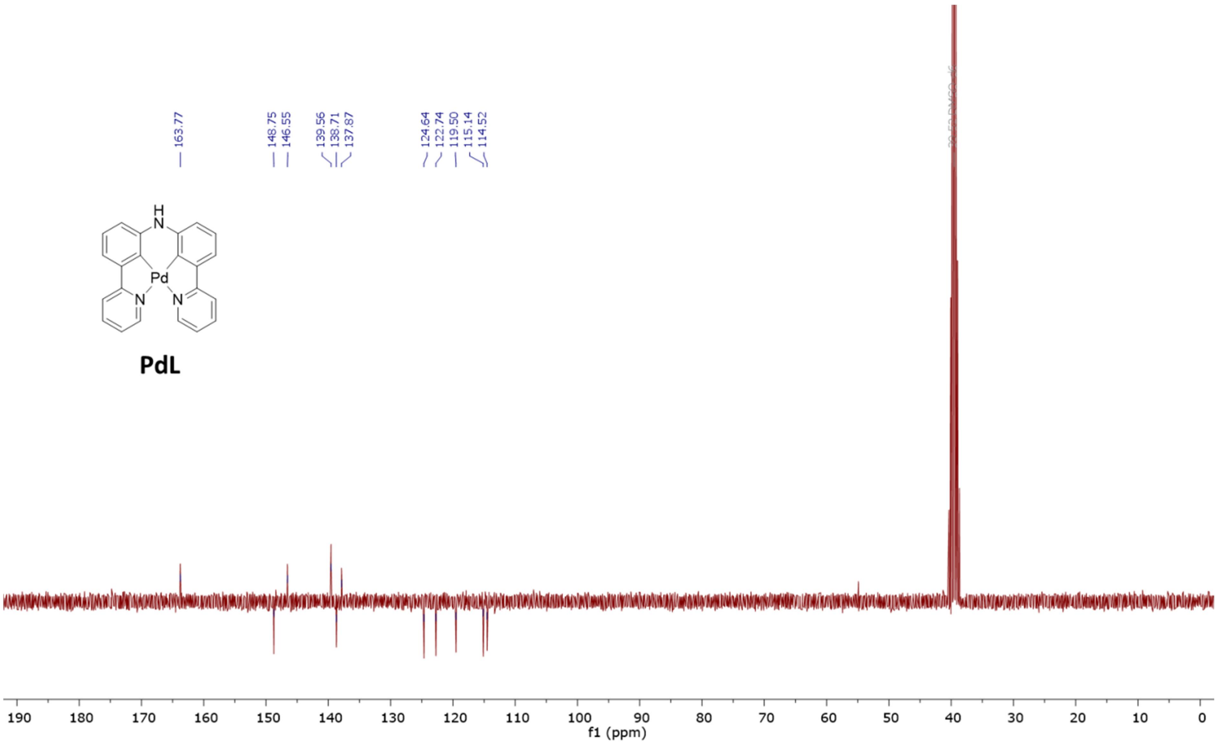

Supplementary Figure 4. ^{13}C-APT NMR of **PdL** in DMSO-d_6.

3. Single crystal X-ray crystallography

All reflection intensities were measured at 110(2) K using a SuperNova diffractometer (equipped with Atlas detector) with Mo *K*α radiation (λ = 0.71073 Å) under the program CrysAlisPro (Version CrysAlisPro 1.171.39.29c, Rigaku OD, 2017). The same program was used to refine the cell dimensions and for data reduction. The structure was solved with the program SHELXS-2018/3 (Sheldrick, 2018) and was refined on F^2 with SHELXL-2018/3 (Sheldrick, 2018). Numerical absorption correction based on gaussian integration over a multifaceted crystal model was applied using CrysAlisPro. The temperature of the data collection was controlled using the system Cryojet (manufactured by Oxford Instruments). The H atoms were placed at calculated positions using the instruction AFIX 43 with isotropic displacement parameters having values 1.2 U_{eq} of the attached C or N atoms. The structure is ordered.

Supplementary Table 1. Crystallographic data for **PdL**.

Complex	**PdL**
Crystal data	
Chemical formula	$C_{22}H_{15}N_3Pd$
M_r	427.77
Crystal system, space group	Monoclinic, $P2_1/n$
Temperature (K)	110
a, *b*, *c* (Å)	12.2301 (5), 10.3563 (4), 12.8772 (4)
β (°)	100.128 (4)
V (Å^3)	1605.59 (11)
Z	4
Radiation type	Mo *K*α
μ (mm^{-1})	1.17
Crystal size (mm)	0.09 × 0.08 × 0.03
Data collection	
Diffractometer	SuperNova, Dual, Cu at zero, Atlas
Absorption correction	Gaussian *CrysAlis PRO* 1.171.39.29c (Rigaku Oxford Diffraction, 2017) Numerical absorption correction based on gaussian integration over a multifaceted crystal model Empirical absorption correction using spherical harmonics, implemented in SCALE3 ABSPACK scaling algorithm.
T_{min}, T_{max}	0.880, 1.000
No. of measured, independent and observed [$I > 2\sigma(I)$] reflections	12313, 3695, 2920
R_{int}	0.048
$(\sin \theta/\lambda)_{max}$ (Å^{-1})	0.650

Refinement	
$R[F^2 > 2\sigma(F^2)]$, $wR(F^2)$, S	0.033, 0.072, 1.03
No. of reflections	3695
No. of parameters	235
H-atom treatment	H-atom parameters constrained
$\Delta\rho_{max}$, $\Delta\rho_{min}$ (e Å^{-3})	0.85, -0.51

Supplementary Table 2. Selected bond distances (Å) and angels (degree) in the crystal structure of **PdL**.

Distance (Å)		Angel (˚)	
Pd-N1	2.144(3)	C11-Pd1-C17	92.09(12)
Pd-C11	1.969(3)	C11-Pd1-N1	80.17(11)
Pd-C17	1.972(3)	C17-Pd1-N1	171.99(11)
Pd-N3	2.163(3)	C17-Pd1-N3	80.24(11)˚
Pd-Pd	3.5176(5)	N1-Pd1-N3	107.42(10)

4. DFT and TDDFT calculation of PdL

Supplementary Table 3. Cartesian coordinates (Å) for the DFT-optimized geometries of a monomer of **PdL**.

Pd	0.67116649	0.92075632	1.31632296
C	1.15114464	2.54713629	-1.36619607
H	2.04650518	1.95233653	-1.45450155
C	0.85734769	3.48393598	-2.33606247
H	1.52447120	3.61600654	-3.17718477
C	-0.30023989	4.22989275	-2.19384479
H	-0.57329940	4.97642763	-2.92956769
C	-1.10433894	4.00804598	-1.09453588
H	-2.01100530	4.58086189	-0.96368203
C	-0.74677527	3.04802226	-0.15434925
C	-1.53482901	2.74635139	1.04160974
C	-2.72373489	3.41619686	1.31933960
H	-3.10512403	4.18332156	0.65819638
C	-3.43114092	3.09264660	2.46699215
H	-4.35859938	3.60366439	2.69744951
C	-2.95730503	2.11686747	3.31930163
H	-3.51311165	1.86304244	4.21680767
C	-1.75954601	1.44189002	3.04059702
C	-1.02508121	1.74794756	1.89078079
C	-0.24971193	-0.32230352	3.91600293
C	-0.09695225	-1.22659663	4.97767102
H	-0.84728910	-1.25586540	5.76197619
C	0.99104508	-2.07295855	5.03144032
H	1.09291203	-2.76592210	5.85838815
C	1.94994132	-2.03641222	4.03055529
H	2.79951834	-2.70456511	4.08349912
C	1.79918728	-1.13923416	2.97644677
C	0.70058809	-0.26513502	2.89193618
C	2.78170598	-1.05583121	1.89476763
C	3.92919109	-1.83829598	1.82937443
H	4.13711214	-2.55869807	2.60727090
C	4.80312962	-1.69365460	0.77117874
H	5.69824961	-2.30082157	0.71412386
C	4.51426938	-0.76211548	-0.21129589
H	5.16290433	-0.60742651	-1.06295599
C	3.35877653	-0.01834336	-0.08322935
H	3.11077704	0.71458188	-0.83451494
N	0.38332773	2.32288959	-0.30115108
N	-1.36574230	0.48373923	3.94769973
H	-1.97605199	0.35380082	4.73835306
N	2.50738670	-0.14836750	0.93261000

Supplementary Table 4. Cartesian coordinates (Å) for the DFT-optimized geometries of a dimer of **PdL**.

Pd	0.614417	0.842622	1.252813
C	1.101120	2.483786	-1.406943
H	1.931826	1.810769	-1.550263
C	0.848425	3.466582	-2.339753
H	1.487273	3.562493	-3.206603
C	-0.249304	4.284131	-2.143218
H	-0.494797	5.062860	-2.855222
C	-1.039972	4.083207	-1.031047
H	-1.912197	4.699725	-0.867262
C	-0.720546	3.077564	-0.127087
C	-1.511190	2.771358	1.064167
C	-2.676188	3.469480	1.365693
H	-3.028237	4.273051	0.732210
C	-3.407495	3.114604	2.486773
H	-4.321240	3.643279	2.732411
C	-2.979099	2.079119	3.290820
H	-3.553984	1.798919	4.168519
C	-1.803367	1.379197	2.989467
C	-1.046265	1.713592	1.864056
C	-0.283923	-0.372222	3.875774
C	-0.104803	-1.233078	4.964974
H	-0.867774	-1.279142	5.736750
C	1.022563	-2.023081	5.060734
H	1.143096	-2.687629	5.908534
C	1.991951	-1.967975	4.074100
H	2.866336	-2.601030	4.150202
C	1.815616	-1.108155	2.993574
C	0.681274	-0.289531	2.868149
C	2.791912	-1.032349	1.907513
C	3.973001	-1.765949	1.875432
H	4.215113	-2.435162	2.688560
C	4.832459	-1.638724	0.804859
H	5.755112	-2.205654	0.774805
C	4.490022	-0.786408	-0.230099
H	5.116829	-0.663283	-1.102261
C	3.300375	-0.096289	-0.136576
H	2.997802	0.559710	-0.937344
N	0.358724	2.291654	-0.320011
N	-1.454977	0.356103	3.847131
H	-2.061316	0.233222	4.642476
N	2.472207	-0.193551	0.900813
Pd	-0.614417	-0.842622	-1.252813
C	-1.101120	-2.483786	1.406943
H	-1.931826	-1.810769	1.550263
C	-0.848425	-3.466582	2.339753
H	-1.487273	-3.562493	3.206603
C	0.249304	-4.284131	2.143218

H	0.494797	-5.062860	2.855222
C	1.039972	-4.083207	1.031047
H	1.912197	-4.699725	0.867262
C	0.720546	-3.077564	0.127087
C	1.511190	-2.771358	-1.064167
C	2.676188	-3.469480	-1.365693
H	3.028237	-4.273051	-0.732210
C	3.407495	-3.114604	-2.486773
H	4.321240	-3.643279	-2.732411
C	2.979099	-2.079119	-3.290820
H	3.553984	-1.798919	-4.168519
C	1.803367	-1.379197	-2.989467
C	1.046265	-1.713592	-1.864056
C	0.283923	0.372222	-3.875774
C	0.104803	1.233078	-4.964974
H	0.867774	1.279142	-5.736750
C	-1.022563	2.023081	-5.060734
H	-1.143096	2.687629	-5.908534
C	-1.991951	1.967975	-4.074100
H	-2.866336	2.601030	-4.150202
C	-1.815616	1.108155	-2.993574
C	-0.681274	0.289531	-2.868149
C	-2.791912	1.032349	-1.907513
C	-3.973001	1.765949	-1.875432
H	-4.215113	2.435162	-2.688560
C	-4.832459	1.638724	-0.804859
H	-5.755112	2.205654	-0.774805
C	-4.490022	0.786408	0.230099
H	-5.116829	0.663283	1.102261
C	-3.300375	0.096289	0.136576
H	-2.997802	-0.559710	0.937344
N	-0.358724	-2.291654	0.320011
N	1.454977	-0.356103	-3.847131
H	2.061316	-0.233222	-4.642476
N	-2.472207	0.193551	-0.900813

Supplementary Table 5. Main low-energy singlet-singlet transitions of **PdL** in the monomeric or dimeric state according to TDDFT calculations.

State	Energy (nm)	Energy (eV)	Oscillator strength (f)	Orbital transition contribution
Monomer	383.0	3.2369	0.1262	HOMO→LUMO 89.9%
	335.2	3.6989	0.3642	HOMO→LUMO+1 84.7%
	304.65	4.0697	0.7603	HOMO-1→LUMO 70.9%
Dimer	540.1232	2.2955	0.0043	HOMO→LUMO 100%
	501.6673	2.4714	0.0484	HOMO→LUMO 96%
	450.6614	2.7512	0.0168	HOMO→LUMO 47.3%<br>HOMO-1→LUMO 36.9%
	400.7565	3.0938	0.0819	HOMO→LUMO+1 86.4%

5. Photophysical properties of PdL

Supplementary Table 6. Photophysical properties of **PdL** in different conditions.

Solvent	λ_{abs}, nm (ε × 10^{-3} M^{-1} cm^{-1})[a]	λ_{em} (nm)[a,b]	lifetime (μs)	φ_P [c]	φ_Δ [d]	Charge transfer rate (μs^{-1}) [c]	Charge recombination rate (μs^{-1}) [c]
DMSO	343 (25.8), 405 (5.2), 481 (3.7)	564	in air 0.295 degassed 29.6	in air 0.0016 degassed 0.0701	-	3.35	0.42
CD_3OD	-	-	-	-	0.09	-	-
THF	347 (22.5), 410 (4.3), 480 (2.9)	540	0.432±0.005		-	-	-
Cell medium	-	-	in air 0.058 degassed 83.5	-	-	-	-

[a] measurement was carried out in aerated DMSO or THF

[b] excitation and concentration: 419 nm, 100 μM.

[c] measurement was carried out in DMSO, using Rhodamine 6G (φ_P = 0.95) as reference.

[d] singlet oxygen generation quantum yield, measured in CD_3OD.

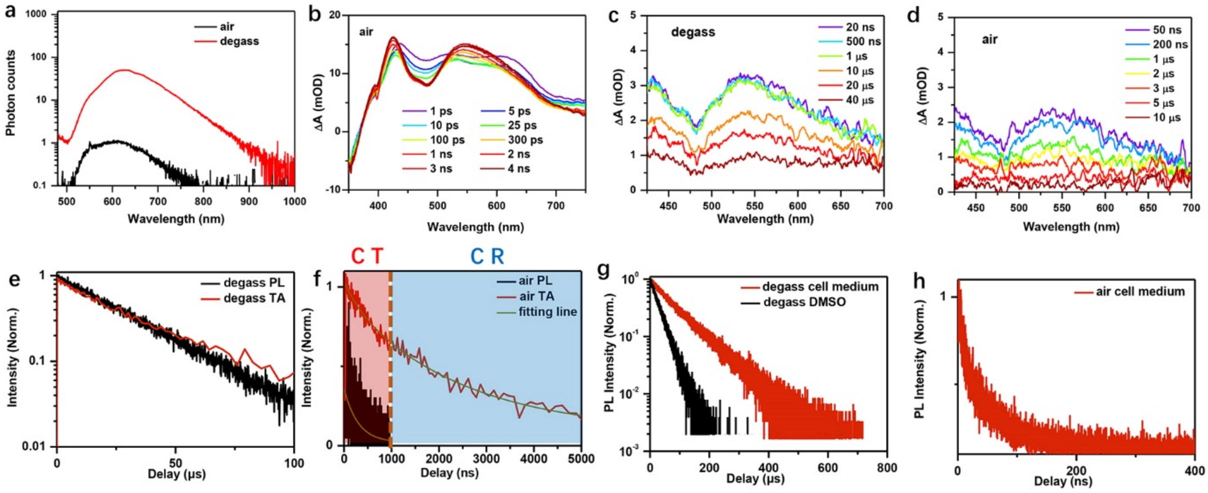

Supplementary Figure 5. (a) Photoluminescence spectra of **PdL** in DMSO under air (black) and degassed (red) condition (excitation: 473 nm). **(b)** Femtosecond transient absorption spectra of **PdL** in DMSO at indicated time delays. **(c, d)** Nanosecond transient absorption spectra of **PdL** in DMSO at indicated time delays under degassed (c) and aerated (d) conditions. **(e, f)** PL kinetics probed at 600 nm (black) and TA kinetics probed at 550 nm (red) under the degassed (e) and aerated (f) conditions. Note: the red and blue regions in (f) represent charge transfer (CT) and charge recombination (CR) processes, respectively. **(g)** PL kinetics probed at 600 nm in cell medium (red) and DMSO (black) under the degassed condition. **(h)** PL kinetics probed at 600 nm in cell medium under air condition. Note: the excitation wavelength of all the TA measurements is 400 nm.

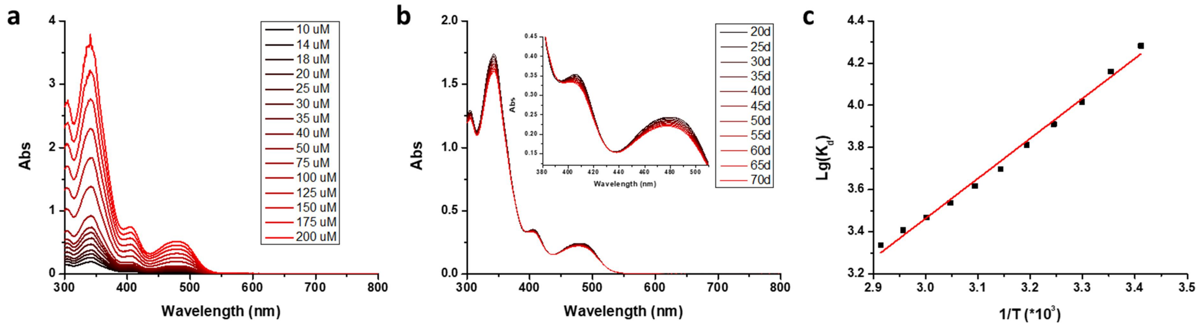

Supplementary Figure 6. (a) Absorption of **PdL** at different concentrations (10-200 μM) in DMSO at room temperature. (b) Absorption of **PdL** (100 μM, DMSO) at different temperatures (20-70 °C). (c) The analyzed data and linear fitting of Log(K_d) with 1/T (Kelvin temperature) of **PdL**.

Supplementary Table 7. Dimerization constant of **PdL** (100 μM, DMSO) at different temperatures.

Temperature (°C)	20	25	30	35	40	45	50	55	60	65	70
K_d (/10^3 M^{-1})	19.1	14.5	10.4	8.1	6.5	5.0	4.1	3.4	2.9	2.6	2.2

6. Self-assembly of PdL in solvent mixtures

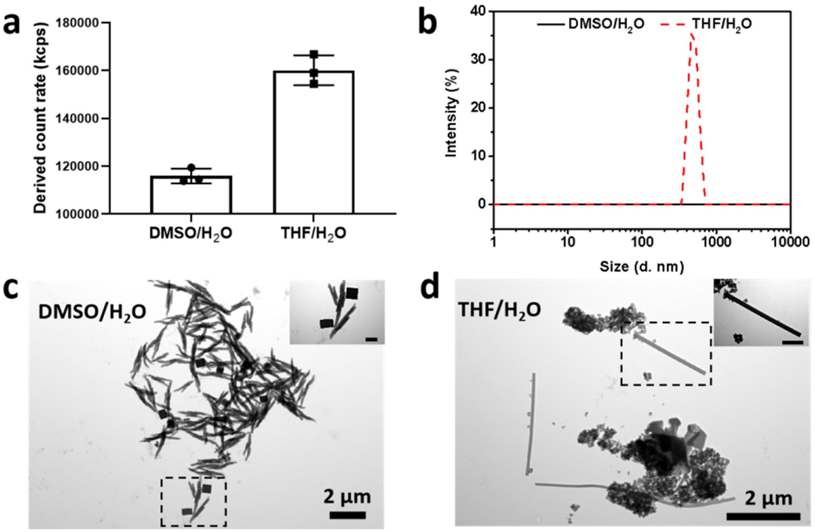

Supplementary Figure 7. (a) DLS derived count rate in the DMSO/H_2O or THF/H_2O system of **PdL** (100 μM) after 30 min self-assembly; data represent mean±s.d. of three replicates. (b) Size distribution of the DLS analysis in the DMSO/H_2O or THF/H_2O system of **PdL** (100 μM) after 30 min self-assembly; TEM images of samples prepared from the DMSO/H_2O (c) or THF/H_2O (d) system of **PdL** (100 μM) after 30 min self-assembly. Inset picture scale bar: 500 nm.

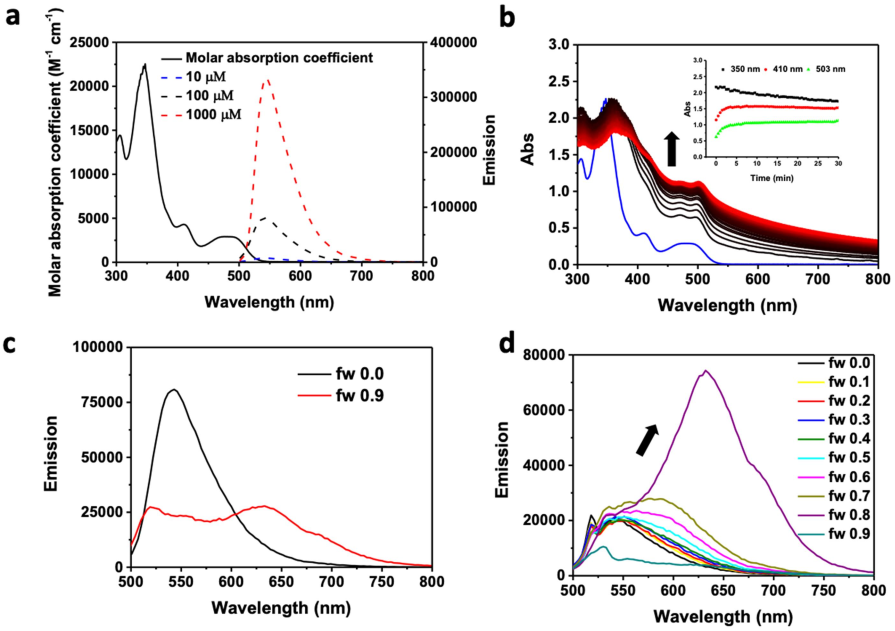

Supplementary Figure 8. (a) The molar absorption coefficient (black solid line) and emission spectra of **PdL** in tetrahydrofuran (THF) solution at different concentrations (blue dash line 10 μM; black dash line 100 μM, red dash line 1000 μM). (b) Time evolution of the absorption spectra of H_2O/THF solution (100 μM, 9:1, v/v) of **PdL** at 298 K for 30 min (30 s

interval, the color of spectra change from black (0 min) to red (30 min); the blue line is the absorbance spectra of **PdL** (100 μM) in pure THF). Inset: time evolution of the absorption at 350 nm (black square), 480 nm (red dot), 504 nm (green triangle) of the solution. (c) Emission spectra of **PdL** (100 μM) in pure THF (fw (V_{water}/V_{total}) = 0.0) and water/THF mixture (9:1, v/v, fw = 0.9); excitation 419 nm. (d) Emission spectra of **PdL** (20 μM) in different THF/water ratio (from v/v = 10/0 to 1/9, excitation 450 nm).

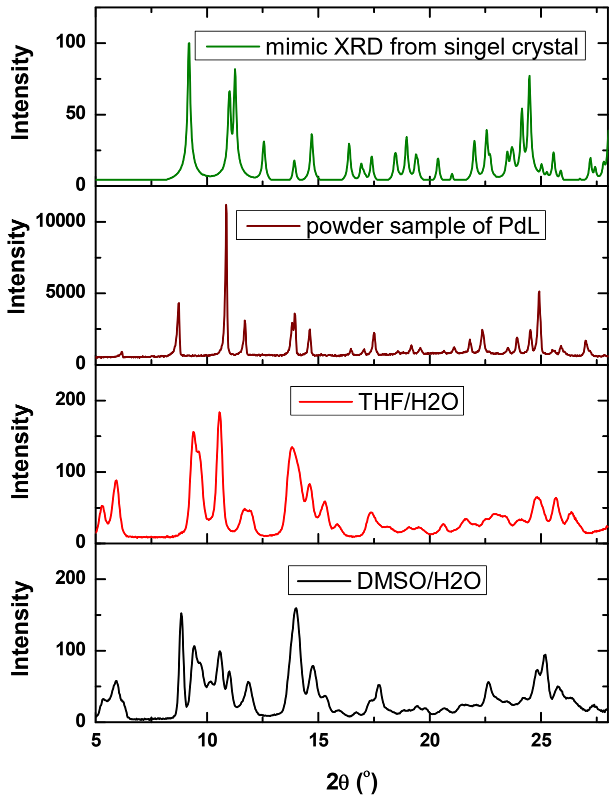

Supplementary Figure 9. Powder XRD pattern of nanoparticles collected from DMSO/H_2O or THF/H_2O (both V/V = 1/9) solution, from a powder sample of **PdL** obtained from synthesis, and calculated PXRD pattern from the crystal structure of **PdL**.

7. Characterization of PdL in Opti-MEM complete medium

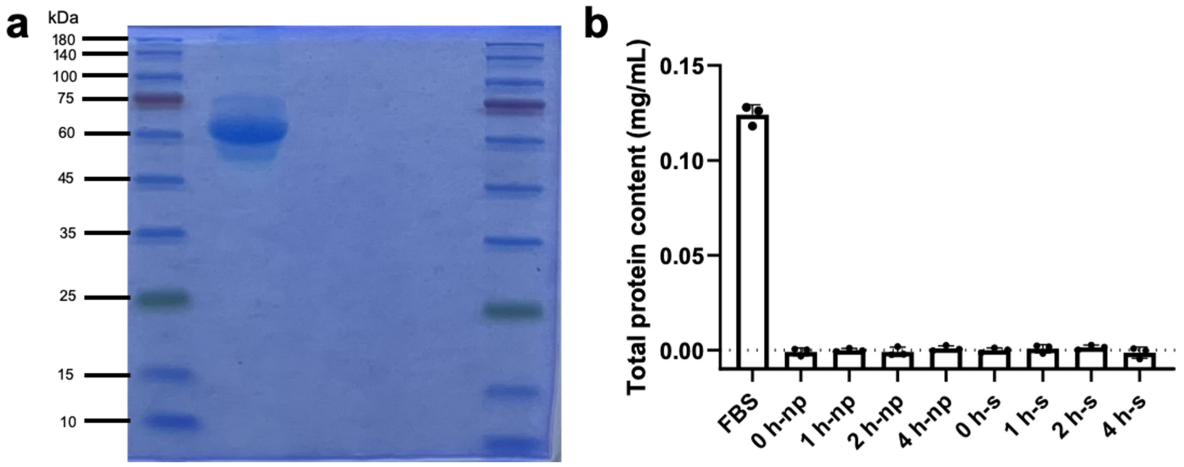

Supplementary Figure 10. (a) Protein gel of marker, fetal bovine serum (FBS), washed **PdL** nanoparticles, supernatant, and marker. (b) Quantification of protein content in FBS, washed **PdL** nanoparticle (np), and supernatant (s) at different reaction time using the Pierce BCA protein assay kit.

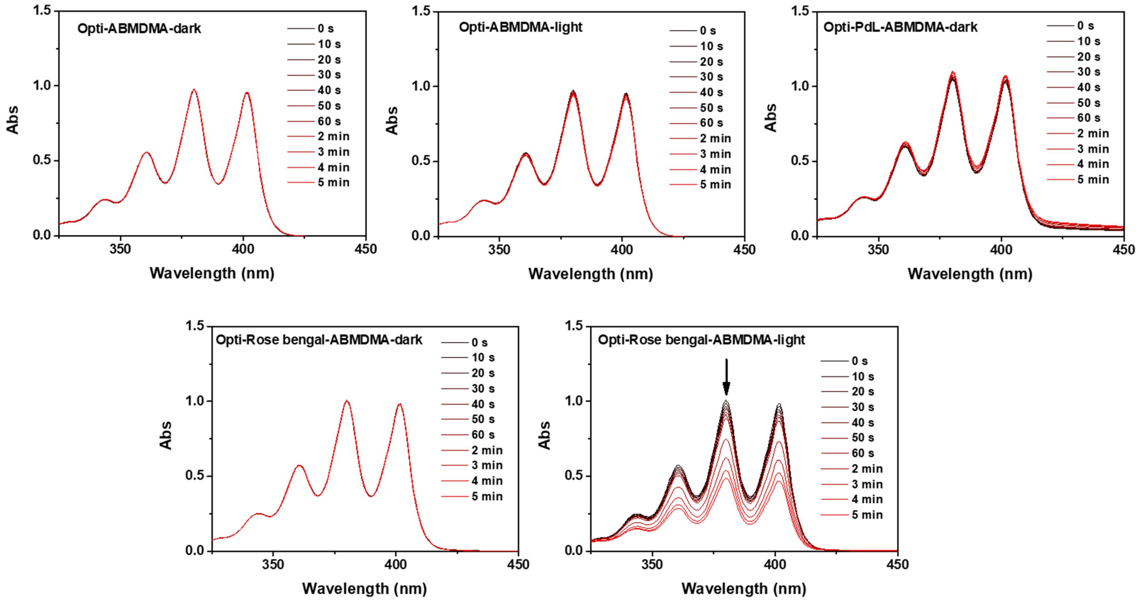

Supplementary Figure 11. Time evolution of the absorption spectrum of an Opti-MEM complete solution of 9,10-anthracenediyl-bis(methylene)-dimalonic acid (ABMDMA, 100 μM) in the absence or presence of **PdL** (25 μM) or rose Bengal, under dark or green light irradiation (515 nm).

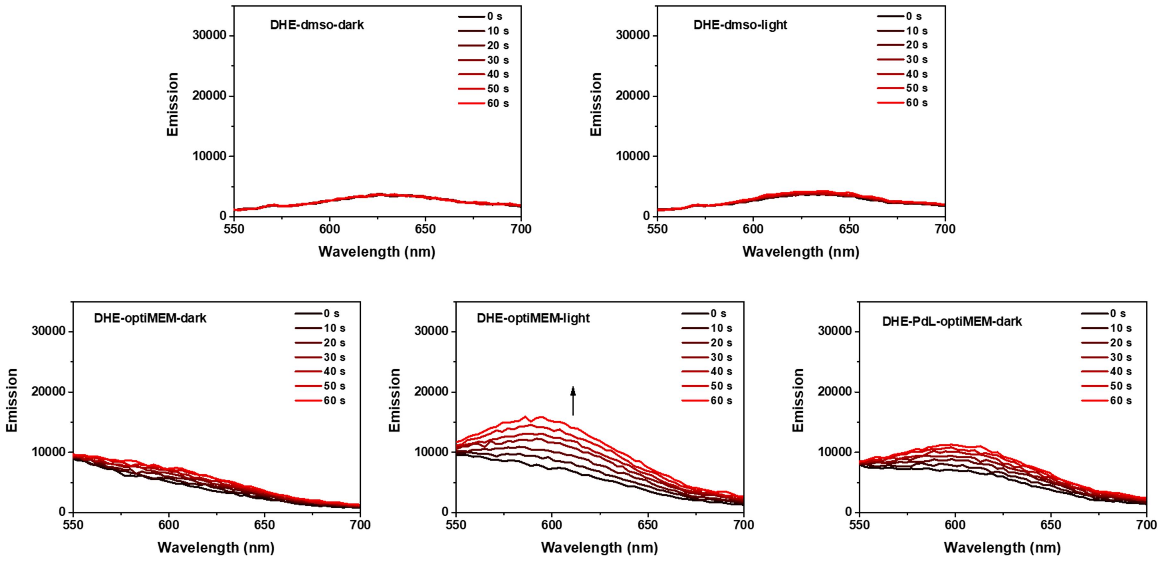

Supplementary Figure 12. The emission spectra of dihydroethidium (DHE) solution (DMSO or Opti-MEM complete) in the absence or presence of **PdL** (25 μM) under green light irradiation (520 nm) or in the dark, over 60 s.

8. Photocytotoxicity of PdL and cell death mechanism determination in cancer cells

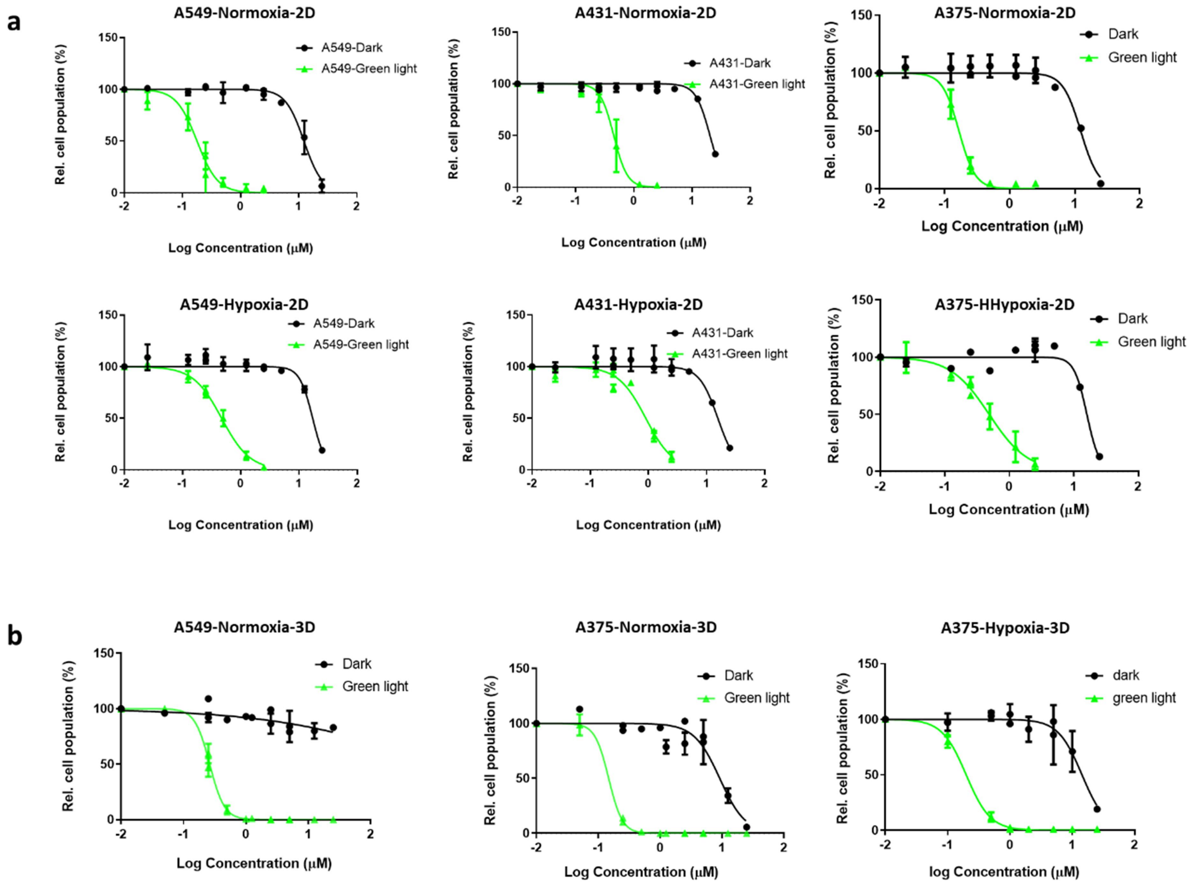

Supplementary Figure 13. Dose-response curves for 2D-monolayer (a) or 3D-spheroid (b) for different human cancer cell lines incubated with **PdL**, either in the dark (black data points) or upon green light irradiation (green data points) under normoxic-2D (520 nm, 20 min, 10.92 mW/cm^2, 13 J/cm^2), hypoxic-2D (520 nm, 32 min, 6.90 mW/cm^2, 13 J/cm^2), normoxia-3D spheroid condition (520 nm, 32 min, 6.90 mW/cm^2, 13 J/cm^2), or hypoxia-3D spheroid condition (520 nm, 55 min, 3.99 mW/cm^2, 13 J/cm^2). Data points are averages (n=3) with 95% confidence intervals (in μM).

Supplementary Table 8. Half-maximal effective concentration (EC_{50} in μM) of **PdL** for A549, A5431 and A375 cancer cells in normoxic, hypoxic or 3D-normoxic and 3D-hypoxic spheroids conditions under dark or green light irradiation. Data are averages (n=3) with 95% confidence intervals (in μM) over three independent experiments. Photoindex (PI = $EC_{50,\ dark}/EC_{50,\ light}$) are also indicated.

Cell line	EC_{50} Values (μM)								
	Condition	2D Normoxic		2D Hypoxic		3D Normoxic		3D Hypoxic	
**A549**	dark	12	+1.3, -1.3	17	+1.5, -1.3	>25		N.D	
	light	0.18	+0.02,-0.02	0.47	+0.04, -0.03	0.26	+0.01, -0.01		
	PI	68		37		>96			
**A431**	dark	20	+1.1, -1.1	16	+1.7, -1.6	N.D		N.D	
	light	0.45	+0.06, -0.05	0.90	+0.1, -0.1				
	PI	45		17					
**A375**	dark	12	+1.3, -1.4	16	+1.6, -1.4	9.2	+2.1, -1.8	14	+4.9, -2.7
	light	0.17	+0.01, -0.01	0.49	+0.09, -0.07	0.17	+0.01, -0.02	0.20	+0.01, -0.01
	PI	72		32		54		72	

Irradiation condition: normoxic 520 nm, 20 min, 10.9 mW/cm^2, 13 J/cm^2; hypoxic 520 nm, 30 min, 7.22 mW/cm^2, 13 J/cm^2; 3D-normoxic 520 nm, 32 min, 6.90 mW/cm^2, 13 J/cm^2; 3D-hypoxic 520 nm, 55 min, 3.99 mW/cm^2, 13.2 J/cm^2. N.D means not determined.

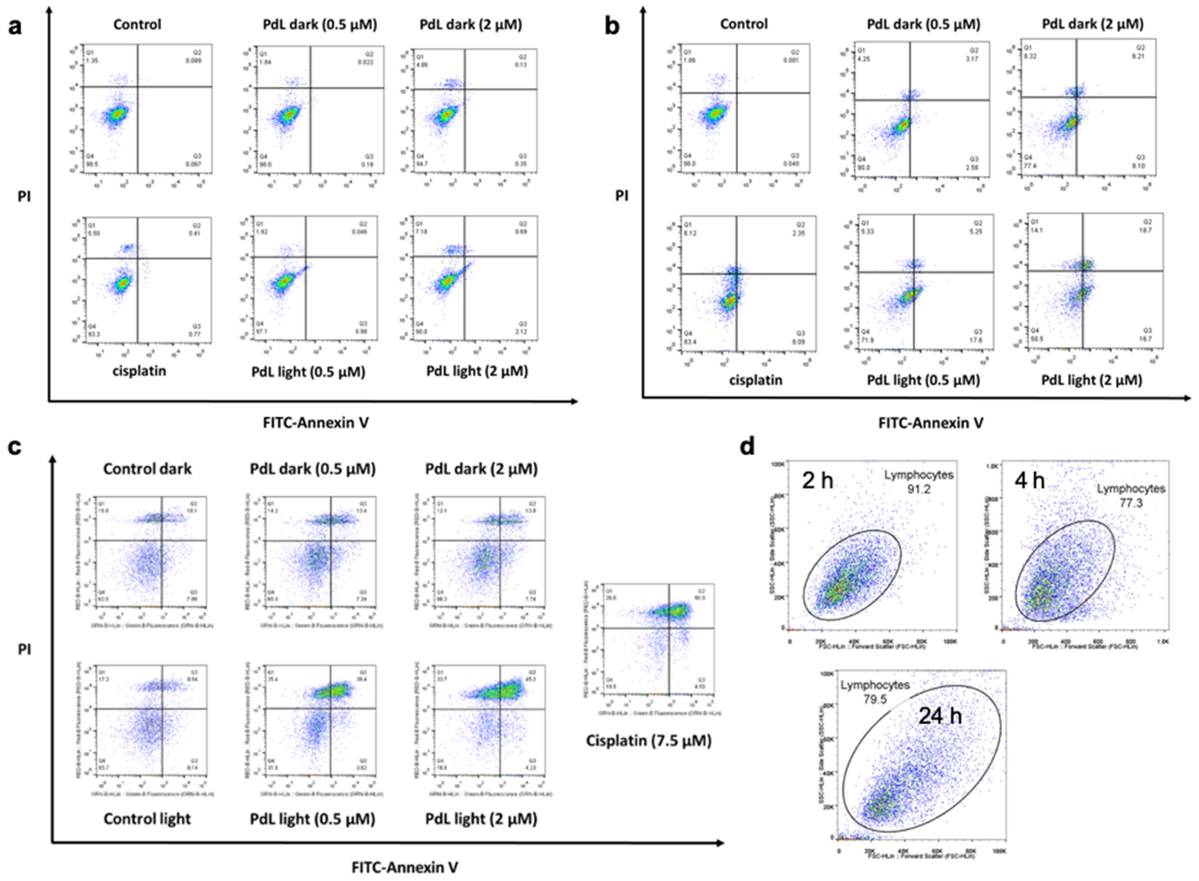

Supplementary Figure 14. Annexin V/Propidium iodide double staining FACS data for A375 cells after treatment with cisplatin (7.5 μM) or **PdL** (0.5 μM or 2 μM) in the dark or upon green light irradiation (normoxic 520 nm, 20 min, 10.9 mW/cm^2, 13 J/cm^2) after 2 (a), 4 (b) and 24 h (c), and the SSC/FSC gating strategy of cells after 2, 4 or 24 h treatments (d).

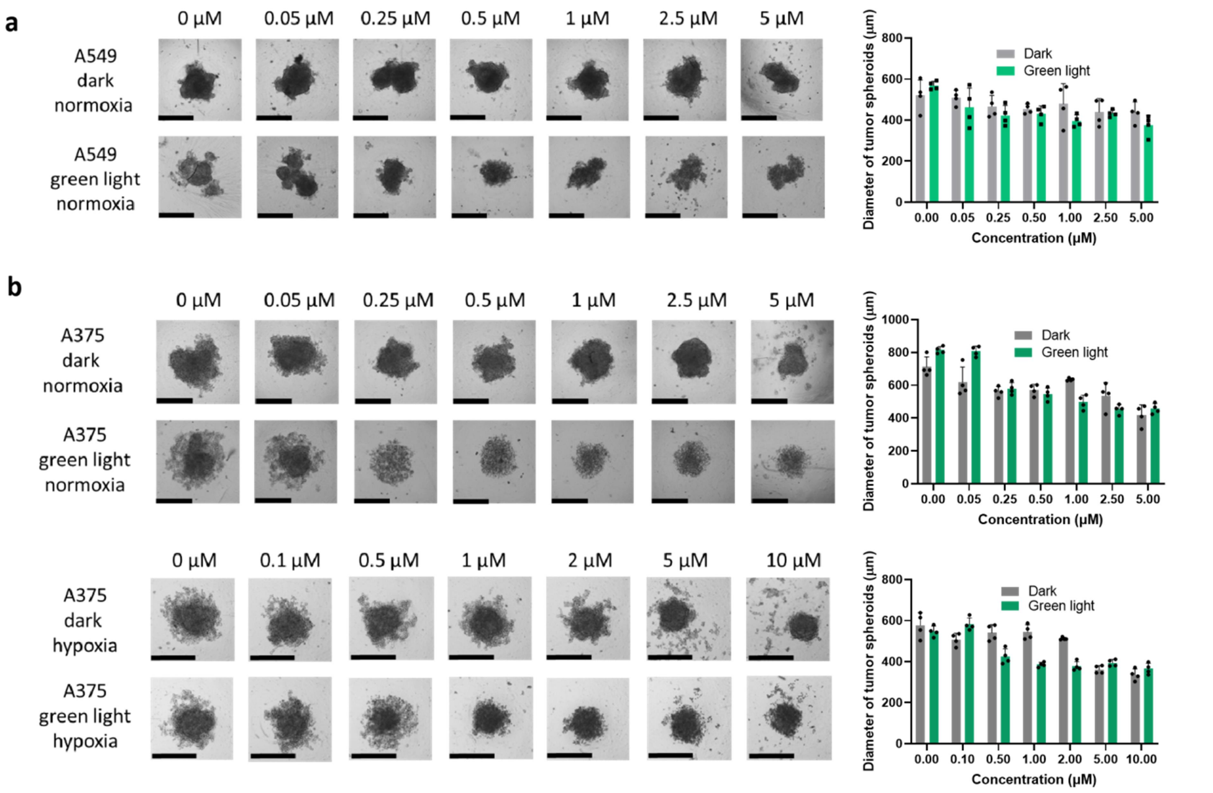

Supplementary Figure 15. Bright filed images (left) and diameter (right, μm) for A549 (a) and A375 (b) 3D tumor spheroids kept in the dark (black bars) or irradiated with green light (green bars, 520 nm, 13 J/cm^2). Scar bar 500 μm. Data represent mean±s.d. of four replicates.

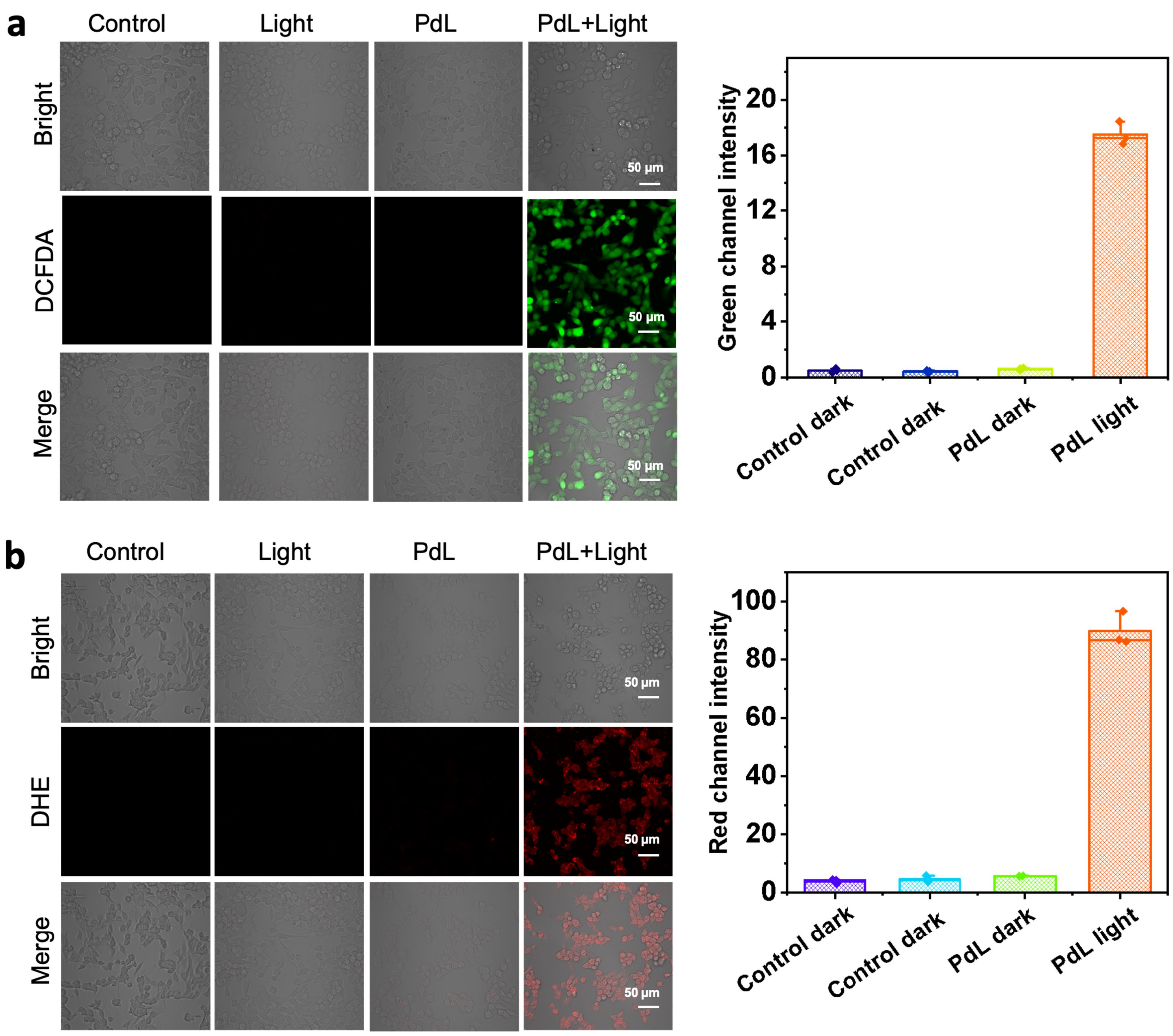

Supplementary Figure 16. Intracellular ROS determination by DCFDA/H_2DCFDA cellular ros assay kit (a), and superoxide radical determination by DHE (b) after treatment with **PdL** (20 μM) under dark or green light irradiation. Data represent mean±s.d. of three replicates.

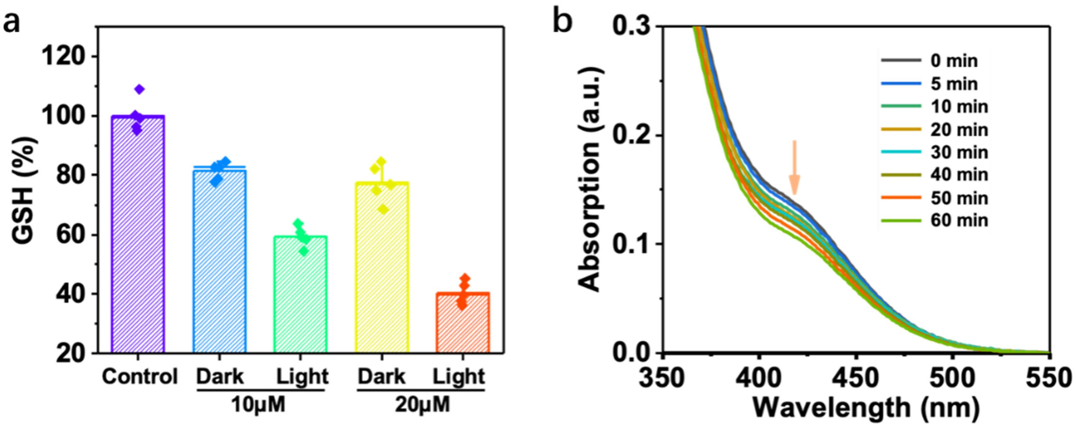

Supplementary Figure 17. (a) Cellular glutathione (GSH) level (%) after treatment with **PdL** in the dark or under light irradiation (520 nm, 10.9 mW/cm^2, 20 min); data represent

mean±s.d. of five biological replicates. (b) UV-vis spectra of **PdL**-GSH-DTNB mixtures in PBS after different green light irradiation times.

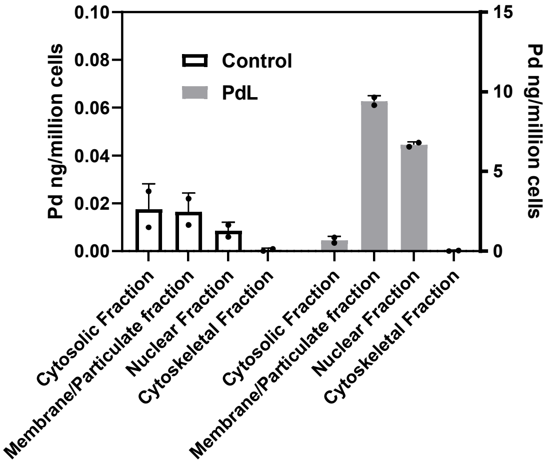

Supplementary Figure 18. Intracellular distribution (expressed in ng Pd/million cells) of **PdL** in the cytosol, membranes, nucleus, and cytoskeleton fractions of A375 cells after treatment by 0 µM (left, light grey) or 5 µM (right, dark grey) for 24 h, as measured by ICP-MS. Data represent mean±s.d. of two biological replicates.

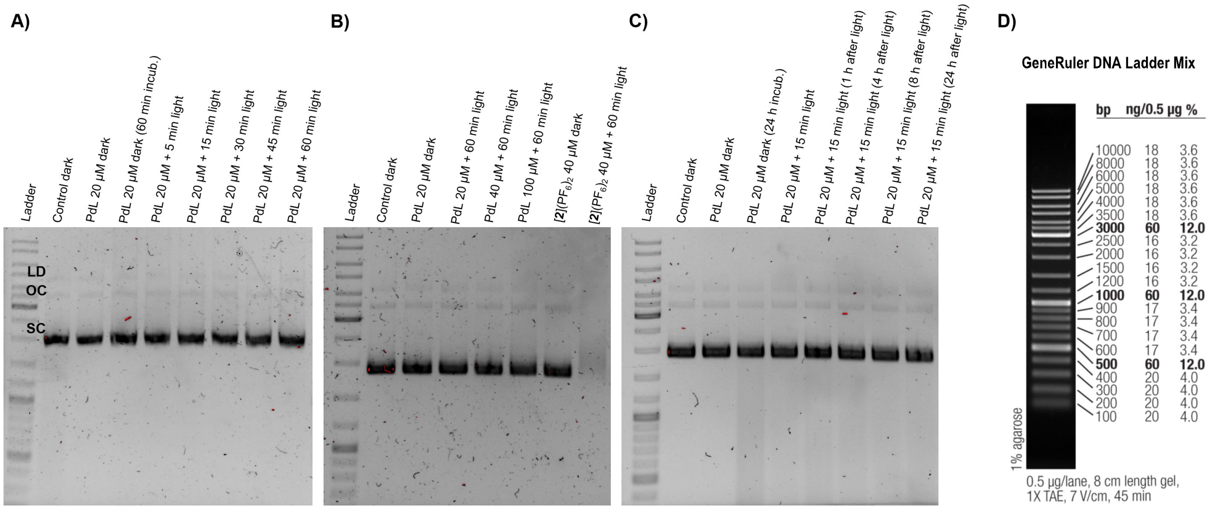

Supplementary Figure 19. Agarose gel of **PdL** in the dark or green light irradiation (520 nm) to pUC19 plasmid DNA at different irradiation times (A), concentrations (B), and incubation times after light activation (C). SC, OC, LD correspond to the supercoiled, open circular, and linear dimer forms of the plasmid, respectively. (D) The commercial DNA marker information.

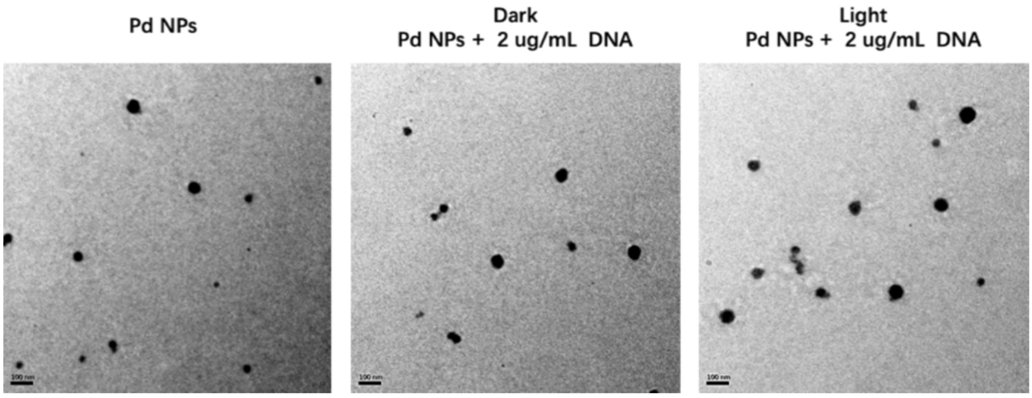

Supplementary Figure 20. The TEM images of Pd nanoparticle (collected from 420 μM of cell medium) and pUC19 DNA (2 mg/mL) PBS solution under dark or green light irradiation (20 min, 10.92 mW/cm^2, 13 J/cm^2) at 37 degrees. Scale bar: 100 nm.

9. Tumor xenografts experiments

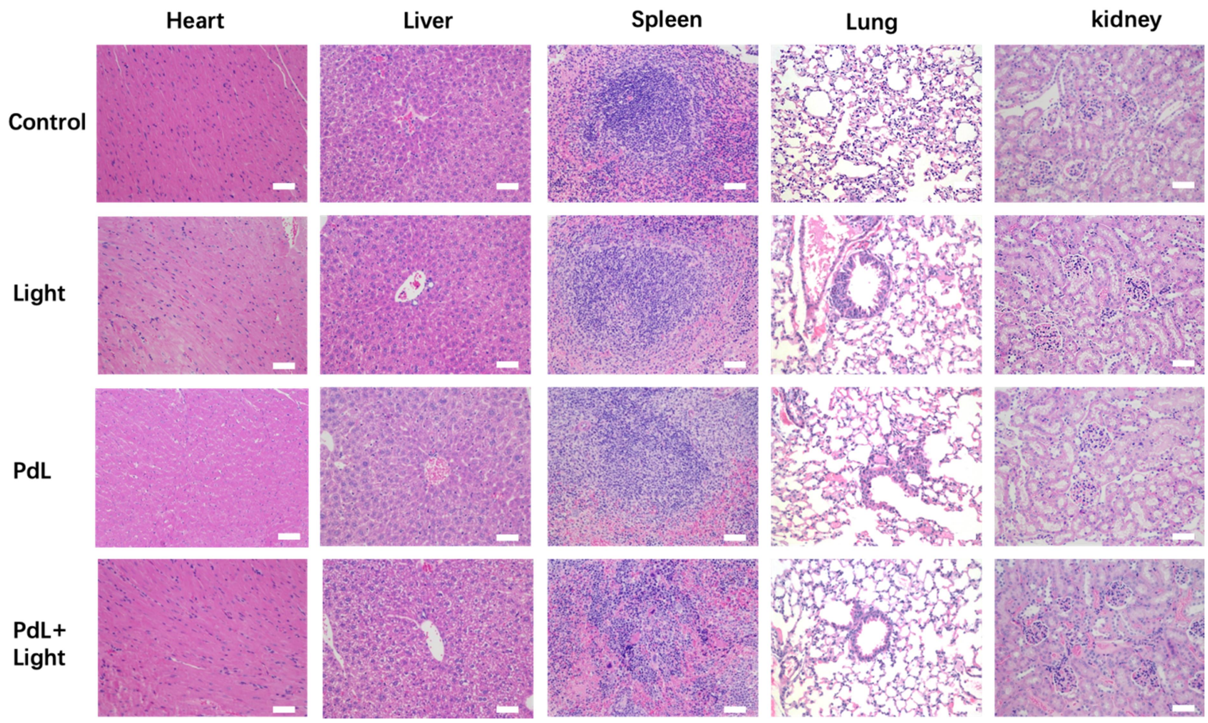

Supplementary Figure 21. The H&E staining of different mice organs after treatment with vehicle control or **PdL**, and either without or with green light irradiation (100 mW/cm^2, 10 min, 60 J/cm^2). Scale bar 200 μm.

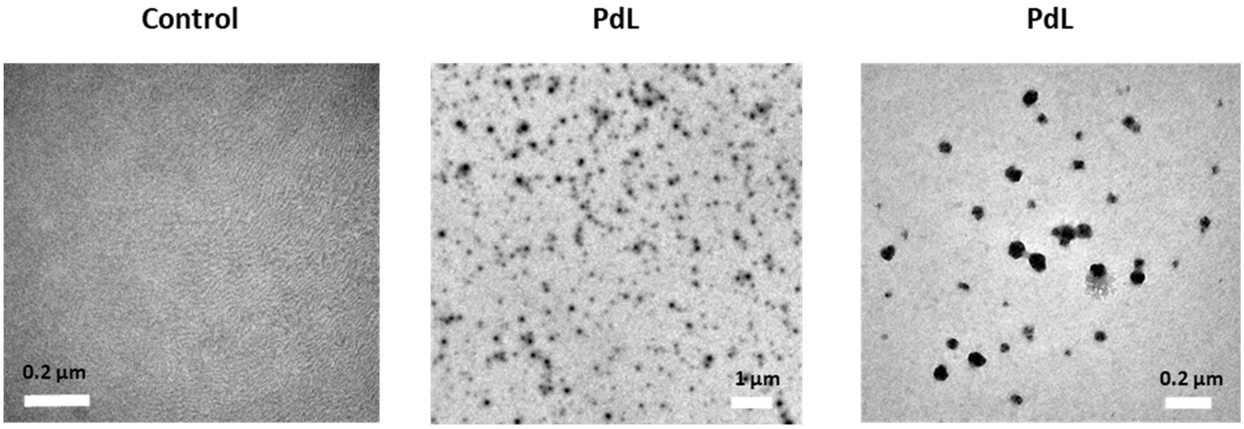

Supplementary Figure 22. EM images showing the morphology of nanoparticles found in the blood of mice 5 min after intravenous tail injection of **PdL** in DMEM medium (middle and right images), or in an untreated control mice (left image). Injection dose: 2.1 μmol/kg, 420 μM, 100 μL DMEM medium (10% FBS), 0.9 mg/kg.

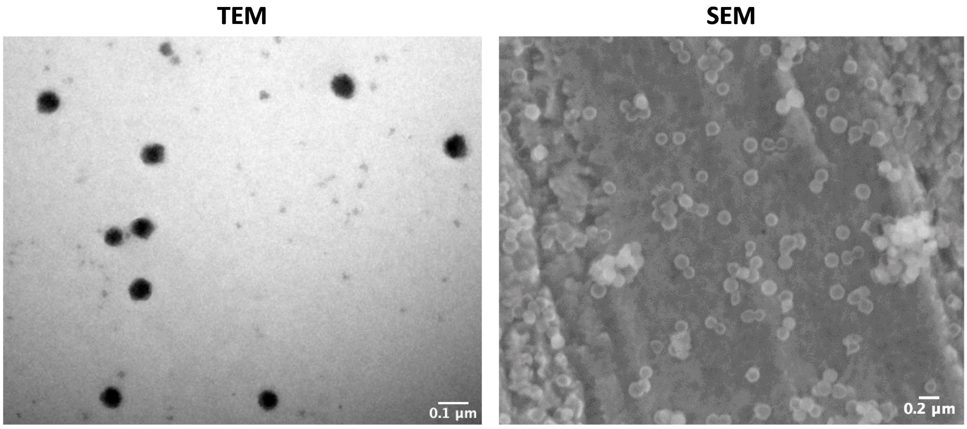

Supplementary Figure 23. TEM and SEM images of nanoparticles collected from mice blood after tail intravenous injection of a DMSO solution of **PdL** (10 µL, 4.2 mM).

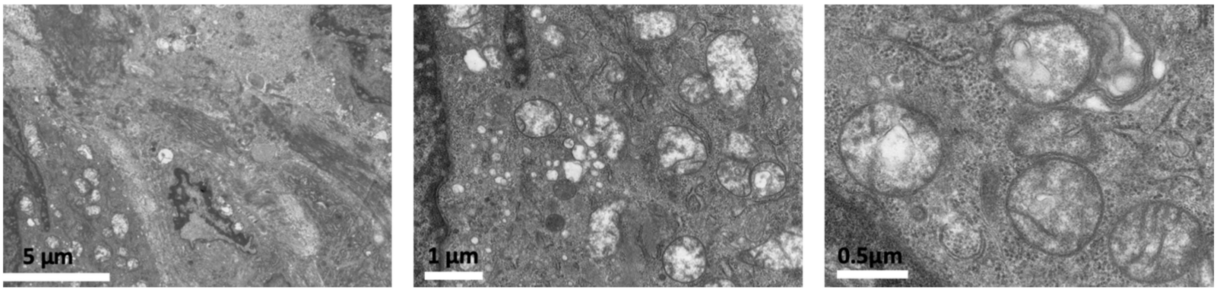

Supplementary Figure 24. EM images at a different magnification of slices of A375 tumor xenografts without the treatment of **PdL**.

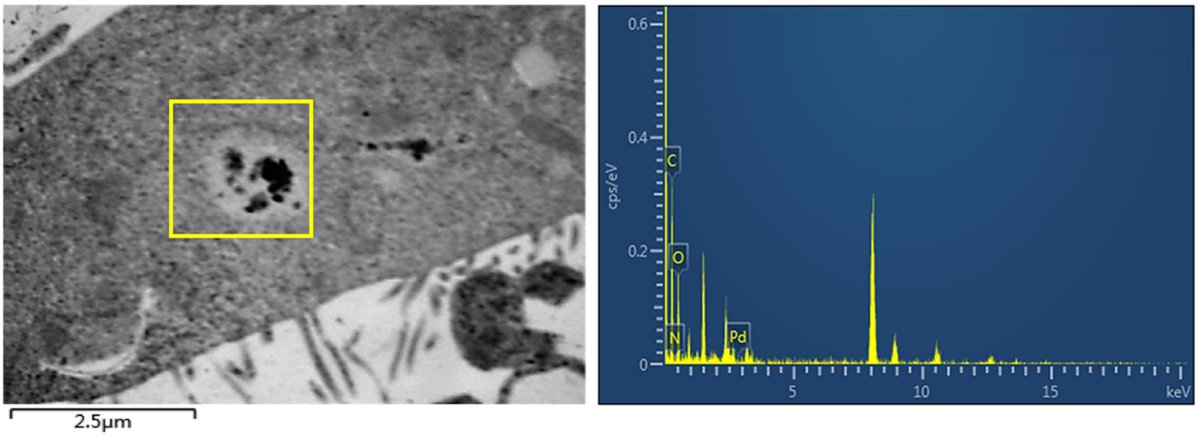

Supplementary Figure 25. TEM images and EDX elemental analysis of nanoparticles found in tumor slices of mice treated with **PdL** for 12 h. Injection dose: 2.1 µmol/kg, 420 µM, 100 µL DMEM medium (10% FBS), 0.9 mg/kg.

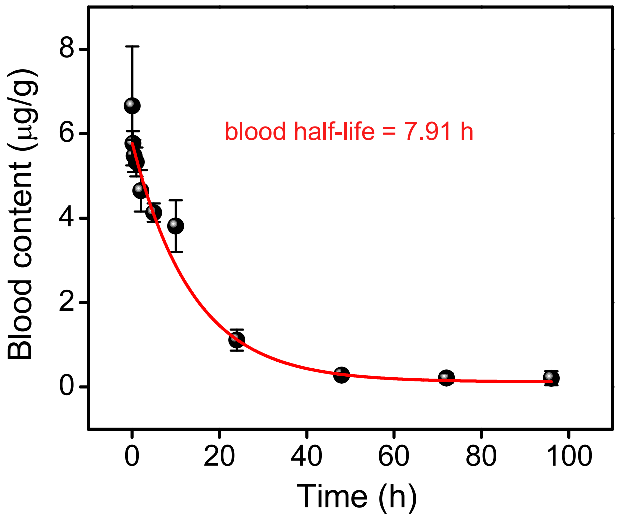

Supplementary Figure 26. *In vivo* blood concentration-time profile of **PdL** following single intravenous injection of drug formulations. The data are presented as means ± s.d. (n = 3).

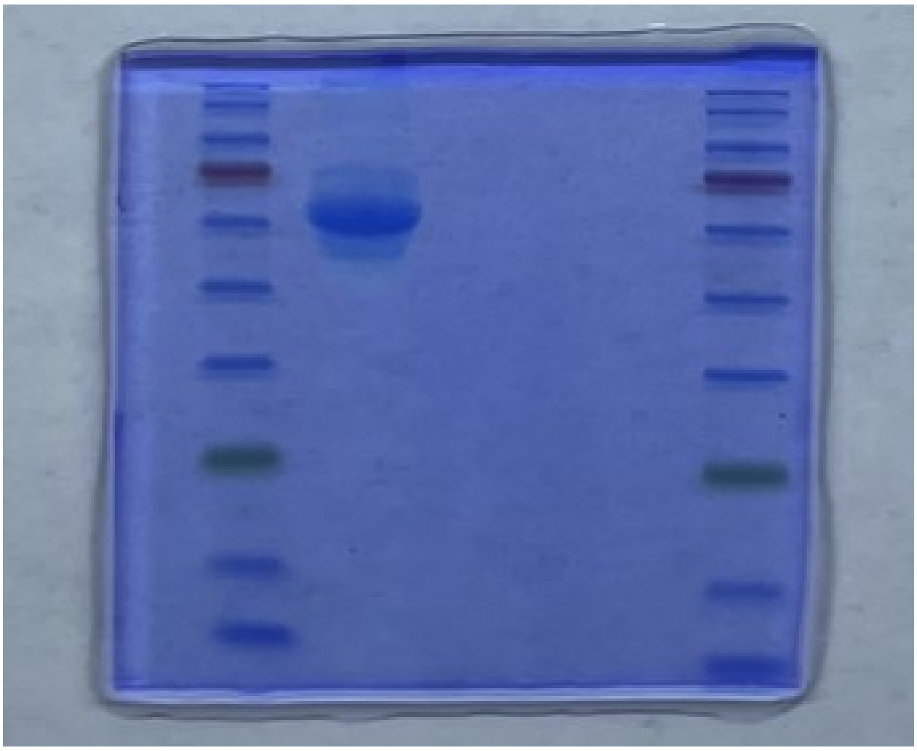

Supplementary Figure 27. Unprocessed protein gel for Supplementary Fig. 10a.

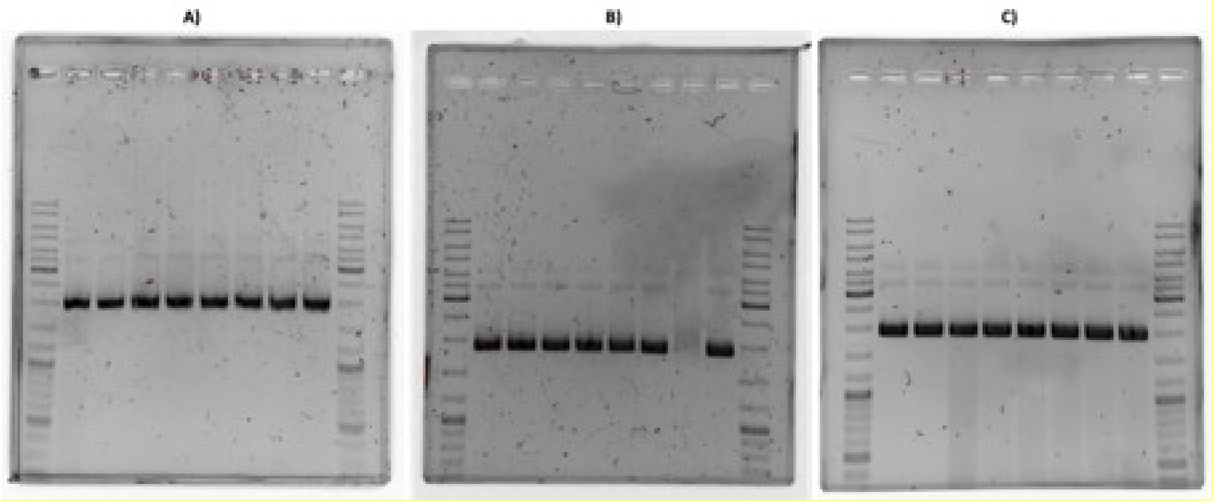

Supplementary Figure 28. Unprocessed DNA gel for Supplementary Fig. 19A-C.

10. References

1. Li, M., *et al.* Brush Conformation of Polyethylene Glycol Determines the Stealth Effect of Nanocarriers in the Low Protein Adsorption Regime. *Nano Lett.* **21**, 1591-1598 (2021).
2. Quanchi, C., *et al.* Photosubstitution in a trisheteroleptic ruthenium complex inhibits conjunctival melanoma growth in a zebrafish orthotopic xenograft model. *Chem. Sci.* **13**, 6899-6919 (2022).
3. Sun, W., *et al.* Biodegradable Drug-Loaded Hydroxyapatite Nanotherapeutic Agent for Targeted Drug Release in Tumors. *ACS Appl. Mater. Interfaces* **10**, 7832-7840 (2018).
4. Antaris, A.L., *et al.* A small-molecule dye for NIR-II imaging. *Nat. Mater.* **15**, 235-242 (2016).
