# Supplementary material for: In vivo metallophilic self-assembly of a light-activated anticancer drug
Source: Nat Chem. 2023 May 11;15(7):980–7. doi: 10.1038/s41557-023-01199-w (PMC10322715; doi:10.1038/s41557-023-01199-w)
Supplement: Supplementary file 2 — Reporting Summary [file 41557_2023_1199_MOESM2_ESM.pdf]

nature portfolio

Corresponding author(s): Wen Sun, Sylvestre Bonnet

Last updated by author(s): Dec 29, 2022

Reporting Summary

Nature Portfolio wishes to improve the reproducibility of the work that we publish. This form provides structure for consistency and transparency in reporting. For further information on Nature Portfolio policies, see our Editorial Policies and the Editorial Policy Checklist.

Statistics

For all statistical analyses, confirm that the following items are present in the figure legend, table legend, main text, or Methods section.

n/a	Confirmed	
☐	☒	The exact sample size (*n*) for each experimental group/condition, given as a discrete number and unit of measurement
☐	☒	A statement on whether measurements were taken from distinct samples or whether the same sample was measured repeatedly
☐	☒	The statistical test(s) used AND whether they are one- or two-sided<br>*Only common tests should be described solely by name; describe more complex techniques in the Methods section.*
☐	☒	A description of all covariates tested
☐	☒	A description of any assumptions or corrections, such as tests of normality and adjustment for multiple comparisons
☐	☒	A full description of the statistical parameters including central tendency (e.g. means) or other basic estimates (e.g. regression coefficient) AND variation (e.g. standard deviation) or associated estimates of uncertainty (e.g. confidence intervals)
☐	☒	For null hypothesis testing, the test statistic (e.g. *F*, *t*, *r*) with confidence intervals, effect sizes, degrees of freedom and *P* value noted<br>*Give P values as exact values whenever suitable.*
☒	☐	For Bayesian analysis, information on the choice of priors and Markov chain Monte Carlo settings
☒	☐	For hierarchical and complex designs, identification of the appropriate level for tests and full reporting of outcomes
☒	☐	Estimates of effect sizes (e.g. Cohen's *d*, Pearson's *r*), indicating how they were calculated

Our web collection on statistics for biologists contains articles on many of the points above.

Software and code

Policy information about availability of computer code

Data collection	Amsterdam Density Functional software (ADF2019)
Data analysis	Origin 8, CCDC, MESTREVONA14.2.2 , Image J 1.52v, GraphPad 8, FlowJo 10

For manuscripts utilizing custom algorithms or software that are central to the research but not yet described in published literature, software must be made available to editors and reviewers. We strongly encourage code deposition in a community repository (e.g. GitHub). See the Nature Portfolio guidelines for submitting code & software for further information.

Data

Policy information about availability of data

All manuscripts must include a data availability statement. This statement should provide the following information, where applicable:

- Accession codes, unique identifiers, or web links for publicly available datasets
- A description of any restrictions on data availability
- For clinical datasets or third party data, please ensure that the statement adheres to our policy

The data relevant with this study are shown in the manuscript and its supplementary Information, or are available in the general repositories Zenodo. Source data are provided with this paper.

March 2021

Human research participants

Policy information about studies involving human research participants and Sex and Gender in Research.

Reporting on sex and gender	N/A
Population characteristics	N/A
Recruitment	N/A
Ethics oversight	N/A

Note that full information on the approval of the study protocol must also be provided in the manuscript.

Field-specific reporting

Please select the one below that is the best fit for your research. If you are not sure, read the appropriate sections before making your selection.

☒ Life sciences ☐ Behavioural & social sciences ☐ Ecological, evolutionary & environmental sciences

For a reference copy of the document with all sections, see nature.com/documents/nr-reporting-summary-flat.pdf

Life sciences study design

All studies must disclose on these points even when the disclosure is negative.

Sample size	No effect size was predetermined, but sample sizes employed in this study are consistent with previously published works (Zhou, Xue-Quan, et al. Journal of the American Chemical Society 142.23 (2020): 10383-10399.). For example, in vitro studies were repeated at least three times independently.
Data exclusions	No data were excluded
Replication	All experiments were repeated for at least three times and experimental findings were reproducible
Randomization	Groups in all the in vitro and in vivo experiments were selected randomly. The dosing groups were filled by randomly selecting from the same pool of animals for in vivo experiments with each group 4 mice. The biological experiments were randomly divided into 4 groups (dark control, 520 nm light, PdL, PdL + 520 nm light groups).
Blinding	All the investigators were blinded to group allocation during data collection and analysis.

Reporting for specific materials, systems and methods

We require information from authors about some types of materials, experimental systems and methods used in many studies. Here, indicate whether each material, system or method listed is relevant to your study. If you are not sure if a list item applies to your research, read the appropriate section before selecting a response.

Materials & experimental systems

n/a	Involved in the study	
☒	☐	Antibodies
☐	☒	Eukaryotic cell lines
☒	☐	Palaeontology and archaeology
☐	☒	Animals and other organisms
☒	☐	Clinical data
☒	☐	Dual use research of concern

Methods

n/a	Involved in the study	
☒	☐	ChIP-seq
☐	☒	Flow cytometry
☒	☐	MRI-based neuroimaging

Eukaryotic cell lines

Policy information about cell lines and Sex and Gender in Research

Cell line source(s)	Human cancer cell lines A549 (lung carcinoma), A431 (skin carcinoma) and A375 (malignant melanoma) were distributed by the European Collection of Cell Cultures (ECACC) and purchased from Sigma Aldrich.

Authentication	A short tandem repeat DNA profiling method was used to authenticate the cell lines
Mycoplasma contamination	All cell lines were tested for mycoplasma contamination. No mycoplasma contamination was found.
Commonly misidentified lines (See ICLAC register)	no commonly misidentified lines are used in this research

Animals and other research organisms

Policy information about studies involving animals; ARRIVE guidelines recommended for reporting animal research, and Sex and Gender in Research

Laboratory animals	Female BALB/c mice with 3 weeks old were originally purchased from Vital River Laboratory Animal Center (Beijing, China). The mice were kept under specific pathogen-free conditions with free access to standard food and water for 2 weeks in 20–21 °C, 40%–60% relative humidity and 12:12 h dark/light cycle, to let the mice weight around 20 g.
Wild animals	the study didn't involve wild animals
Reporting on sex	the study didn't involve reporting on sex
Field-collected samples	The study did not involve samples collected from field.
Ethics oversight	This study was conducted following the Guide for the Care and Use of Laboratory Animals published by the US National Institutes of Health (8th edition, 2011). All protocols for animal studies conformed to the Guide for the Care and Use of Laboratory Animals. All animal experiments were performed under guidelines approved by the ethics committee of Dalian University of Technology.

Note that full information on the approval of the study protocol must also be provided in the manuscript.

Flow Cytometry

Plots

Confirm that:

☒ The axis labels state the marker and fluorochrome used (e.g. CD4-FITC).

☒ The axis scales are clearly visible. Include numbers along axes only for bottom left plot of group (a 'group' is an analysis of identical markers).

☒ All plots are contour plots with outliers or pseudocolor plots.

☒ A numerical value for number of cells or percentage (with statistics) is provided.

Methodology

Sample preparation	incubated with Opti-MEM medium for lysis and washed with PBS. Single-cell suspensions were obtained and stained with Annexin V/propidium iodide dyes for 15 min according to the manufacturer's protocols, and then analyzed by flow cytometry.
Instrument	Guava® easyCyte™
Software	FlowJo
Cell population abundance	The absolute cells around 10000 were analyzed for fluorescent intensity in the defined gate.
Gating strategy	In general, cells were first gated on FSC/SSC. Singlet cells were gated using FSC-H and FSC-A. Dead cells were then excluded. Parameter "GRN-B" (488 nm excitation, 525±30 nm emission) "RED-B" (488 nm excitation, 661±15 nm emission) were used for fluorescence measurements to match with the known excitation/emission wavelengths of Annexin V-FITC (494/518 nm) and propidium iodide (535/617 nm).

☒ Tick this box to confirm that a figure exemplifying the gating strategy is provided in the Supplementary Information.
