# Supplementary material for: In vivo metallophilic self-assembly of a light-activated anticancer drug
Source: Nat Chem. 2023 May 11;15(7):980–7. doi: 10.1038/s41557-023-01199-w (PMC10322715; doi:10.1038/s41557-023-01199-w)

checkCIF/PLATON report

You have not supplied any structure factors. As a result the full set of tests cannot be run.

THIS REPORT IS FOR GUIDANCE ONLY. IF USED AS PART OF A REVIEW PROCEDURE FOR PUBLICATION, IT SHOULD NOT REPLACE THE EXPERTISE OF AN EXPERIENCED CRYSTALLOGRAPHIC REFEREE.

No syntax errors found. CIF dictionary Interpreting this report

Datablock: PdL

```
Bond precision:   C-C = 0.0047 A           Wavelength=0.71073

Cell:              a=12.2301(5)     b=10.3563(4)        c=12.8772(4)
                   alpha=90         beta=100.128(4)     gamma=90
Temperature:       110 K

                  Calculated                   Reported
Volume            1605.59(11)                  1605.59(11)
Space group       P 21/n                       P 21/n
Hall group        -P 2yn                       -P 2yn
Moiety formula    C22 H15 N3 Pd                C22 H15 N3 Pd
Sum formula       C22 H15 N3 Pd                C22 H15 N3 Pd
Mr                427.77                       427.77
Dx,g cm-3         1.770                        1.770
Z                 4                            4
Mu (mm-1)         1.166                        1.166
F000              856.0                        856.0
F000'             852.24
h,k,lmax          15,13,16                     15,13,16
Nref              3693                         3695
Tmin,Tmax         0.900,0.966                  0.880,1.000
Tmin'             0.900

Correction method= # Reported T Limits: Tmin=0.880 Tmax=1.000
AbsCorr = GAUSSIAN

Data completeness= 1.001           Theta(max)= 27.499

R(reflections)= 0.0334( 2920)                          wR2(reflections)=
                                                       0.0719( 3695)

S = 1.032                    Npar= 235
```

The following ALERTS were generated. Each ALERT has the format
test-name_ALERT_alert-type_alert-level.
Click on the hyperlinks for more details of the test.

Alert level C

```
PLAT420_ALERT_2_C D-H Bond Without Acceptor  N2       --H2A      .     Please Check
```

Alert level G

```
PLAT007_ALERT_5_G Number of Unrefined Donor-H Atoms .............          1 Report
PLAT012_ALERT_1_G N.O.K.   _shelx_res_checksum Found in CIF ......     Please Check
PLAT794_ALERT_5_G Tentative Bond Valency for Pd1       (II)      .       1.82 Info
PLAT941_ALERT_3_G Average HKL Measurement Multiplicity ..........         3.3 Low
PLAT965_ALERT_2_G The SHELXL WEIGHT Optimisation has not Converged     Please Check
PLAT967_ALERT_5_G Note: Two-Theta Cutoff Value in Embedded .res ..       55.0 Degree
```

0 **ALERT level A** = Most likely a serious problem - resolve or explain
0 **ALERT level B** = A potentially serious problem, consider carefully
1 **ALERT level C** = Check. Ensure it is not caused by an omission or oversight
6 **ALERT level G** = General information/check it is not something unexpected

1 ALERT type 1 CIF construction/syntax error, inconsistent or missing data
2 ALERT type 2 Indicator that the structure model may be wrong or deficient
1 ALERT type 3 Indicator that the structure quality may be low
0 ALERT type 4 Improvement, methodology, query or suggestion
3 ALERT type 5 Informative message, check

Validation response form

Please find below a validation response form (VRF) that can be filled in and pasted into your CIF.

```
# start Validation Reply Form
_vrf_PLAT420_PdL
;
PROBLEM: D-H Bond Without Acceptor  N2       --H2A      .     Please Check
RESPONSE: ...
;
# end Validation Reply Form
```

It is advisable to attempt to resolve as many as possible of the alerts in all categories. Often the minor alerts point to easily fixed oversights, errors and omissions in your CIF or refinement strategy, so attention to these fine details can be worthwhile. In order to resolve some of the more serious problems it may be necessary to carry out additional measurements or structure refinements. However, the purpose of your study may justify the reported deviations and the more serious of these should normally be commented upon in the discussion or experimental section of a paper or in the "special_details" fields of the CIF. checkCIF was carefully designed to identify outliers and unusual parameters, but every test has its limitations and alerts that are not important in a particular case may appear. Conversely, the absence of alerts does not guarantee there are no aspects of the results needing attention. It is up to the individual to critically assess their own results and, if necessary, seek expert advice.

Publication of your CIF in IUCr journals

A basic structural check has been run on your CIF. These basic checks will be run on all CIFs submitted for publication in IUCr journals (*Acta Crystallographica*, *Journal of Applied Crystallography*, *Journal of Synchrotron Radiation*); however, if you intend to submit to *Acta Crystallographica Section C* or *E* or *IUCrData*, you should make sure that full publication checks are run on the final version of your CIF prior to submission.

Publication of your CIF in other journals

Please refer to the *Notes for Authors* of the relevant journal for any special instructions relating to CIF submission.

PLATON version of 28/11/2022; check.def file version of 28/11/2022

Datablock PdL - ellipsoid plot

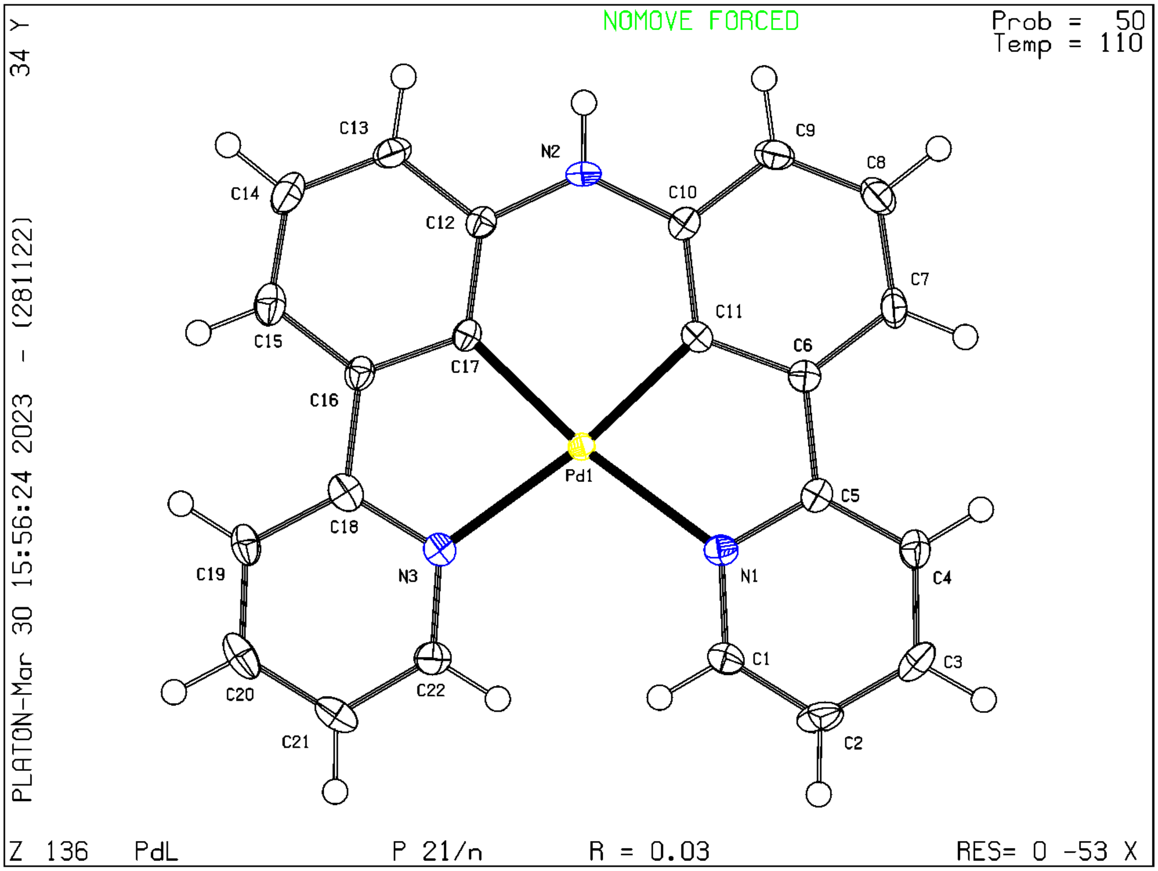

Supplement: Supplementary file 4 — CheckCIF file for the X-ray structure of PdL. [file 41557_2023_1199_MOESM4_ESM.pdf]
