# Supplementary figures and images for: In vivo metallophilic self-assembly of a light-activated anticancer drug
Source: Nat Chem. 2023 May 11;15(7):980–7. doi: 10.1038/s41557-023-01199-w (PMC10322715; doi:10.1038/s41557-023-01199-w)

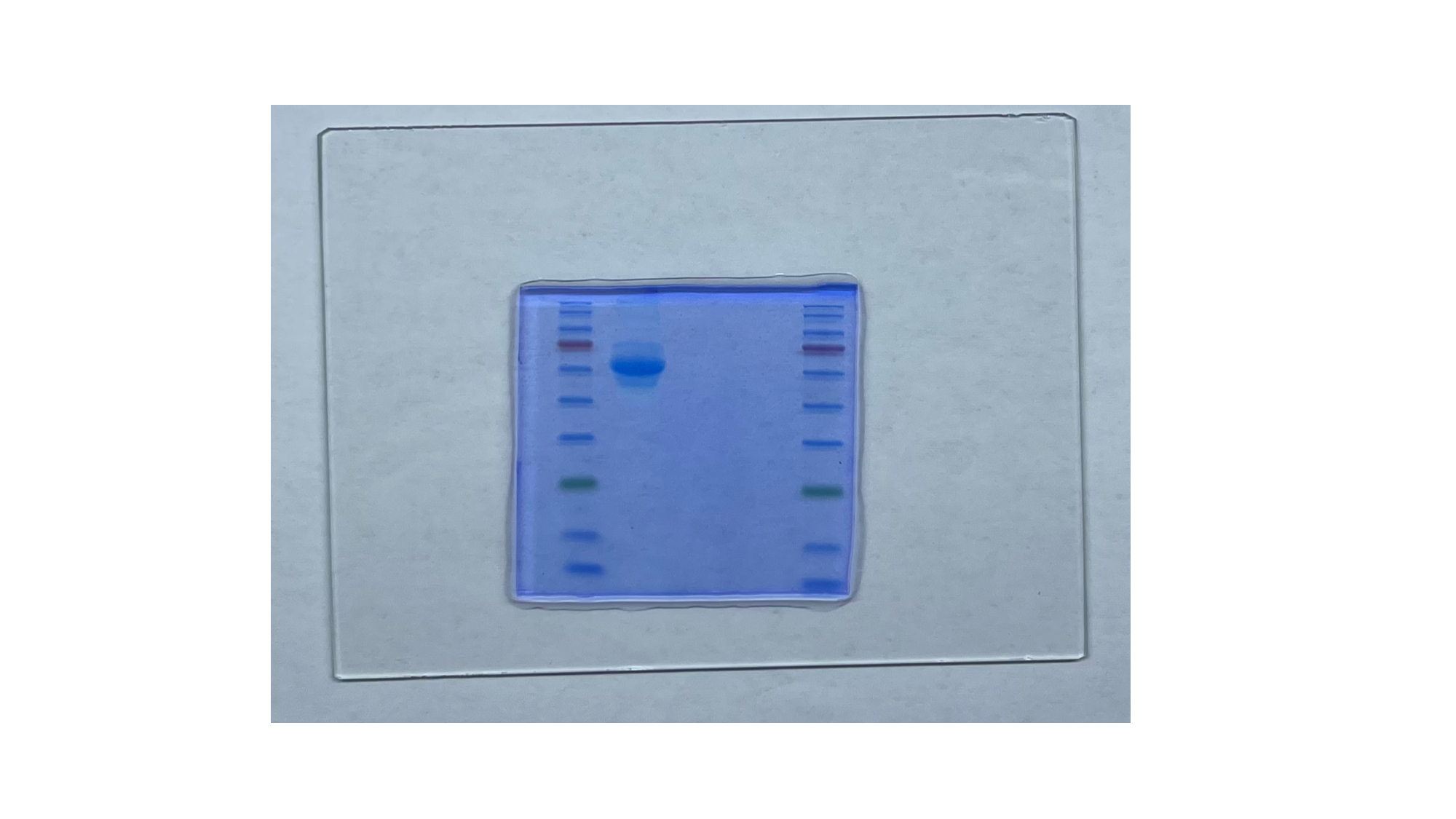

Supplement: Supplementary file 5 — Unprocessed protein gel for Supplementary Fig. 10. [file 41557_2023_1199_MOESM5_ESM.tiff]
