# Supplementary material for: In vivo metallophilic self-assembly of a light-activated anticancer drug
Source: Nat Chem. 2023 May 11;15(7):980–7. doi: 10.1038/s41557-023-01199-w (PMC10322715; doi:10.1038/s41557-023-01199-w)
Supplement: Supplementary file 6 — Unprocessed DNA gel for Supplementary Fig. 19. [file 41557_2023_1199_MOESM6_ESM.pdf]

12/07/2022

PdL 20 µM

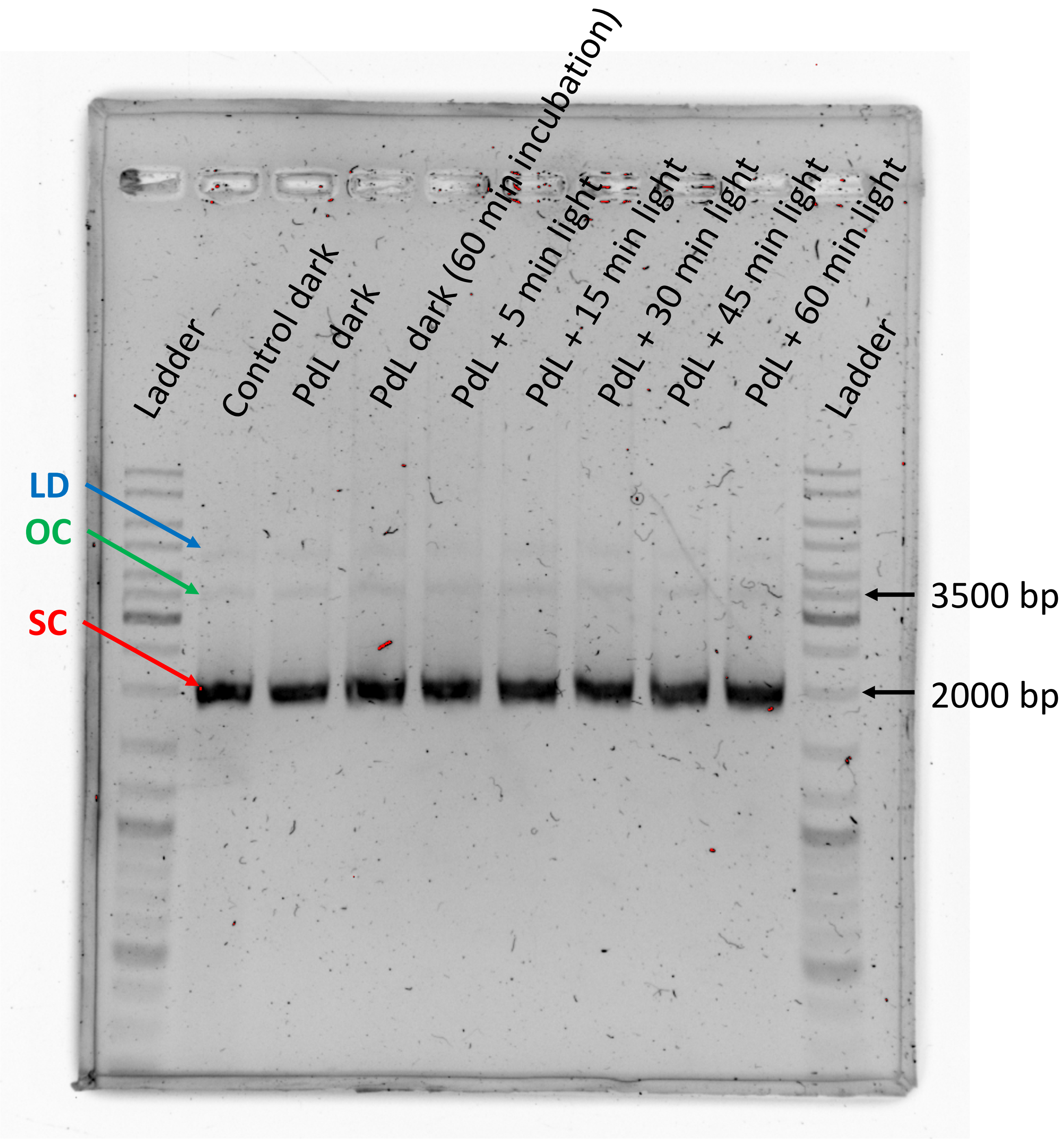

GeneRuler DNA Ladder Mix

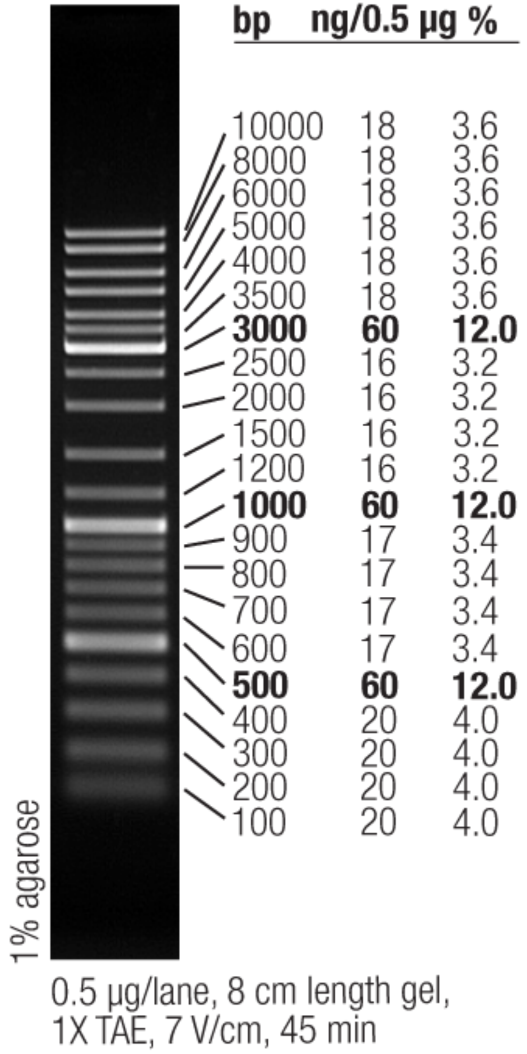

15/07/2022

60 min light activation

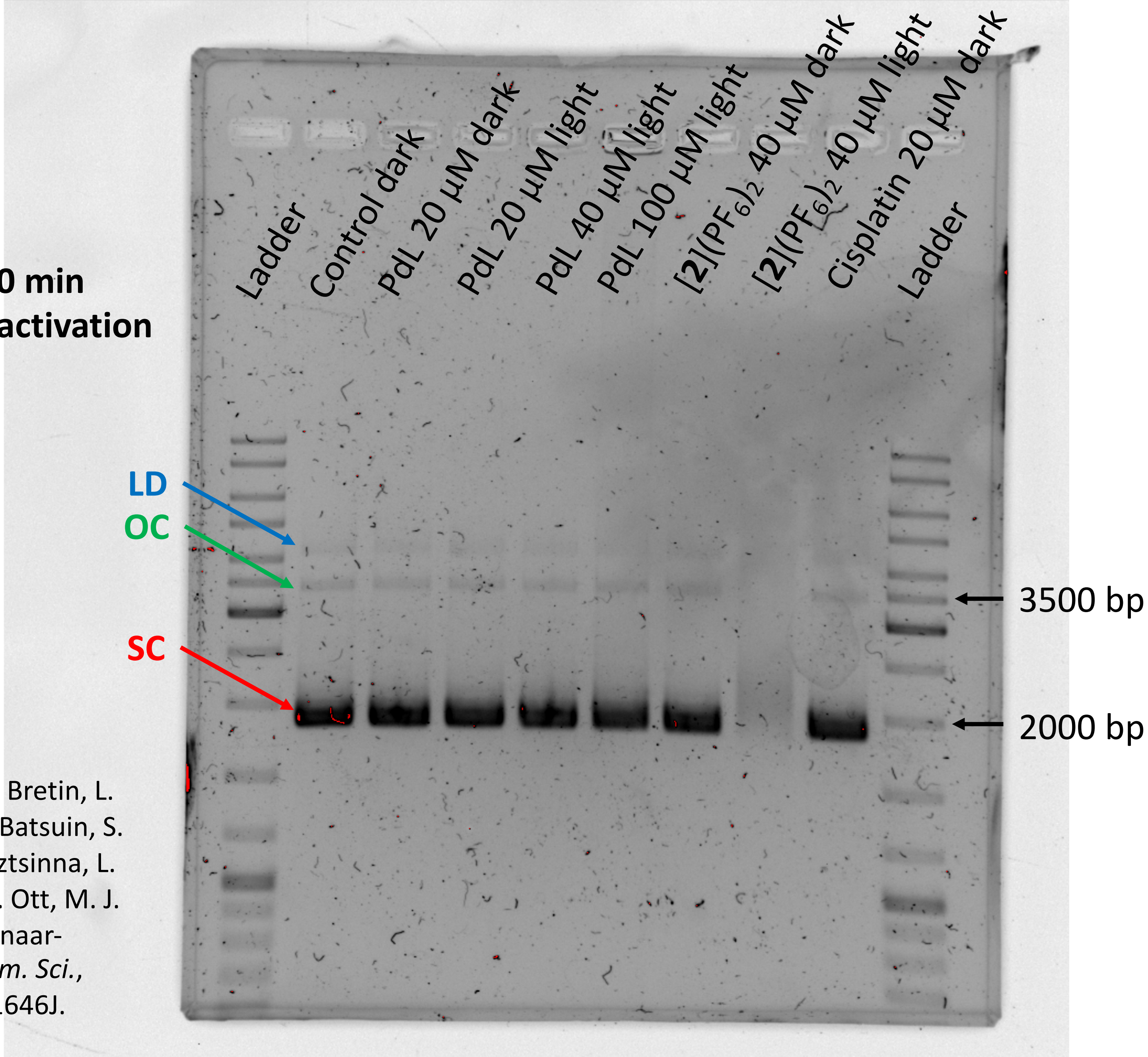

GeneRuler DNA Ladder Mix

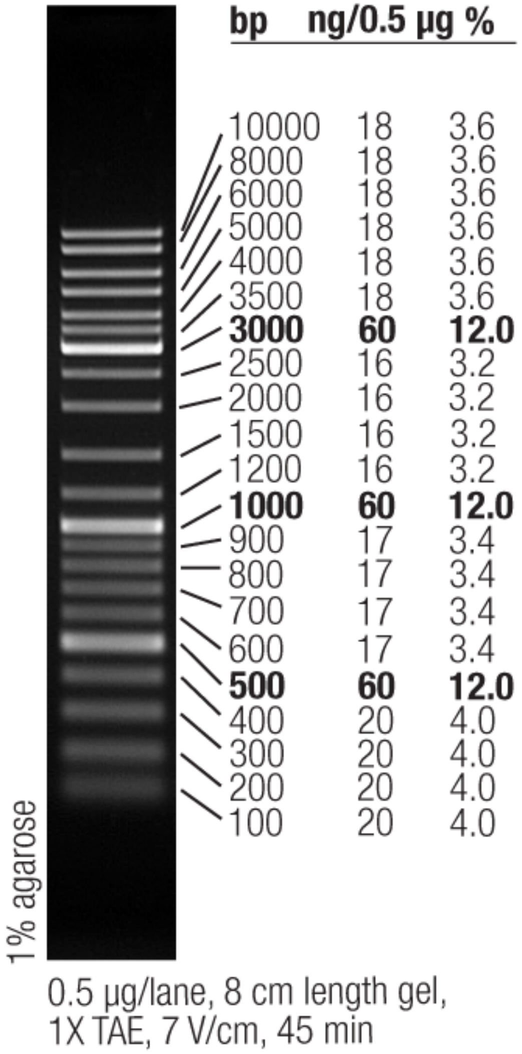

$[\mathbf{2}](PF_6)_2$ = YB32

Q. Chen, J. Cuello-Garibo, L. Bretin, L. Zhang, V. Ramu, Y. Aydar, Y. Batsuin, S. Bronkhorst, Y. Husiev, N. Beztsinna, L. Chen, X. Zhou, C. Schmidt, I. Ott, M. J. Jager, A. M. Brouwer, B. E. Snaar-Jagalska and S. Bonnet, *Chem. Sci.*, 2022, DOI: 10.1039/D2SC01646J.

19/07/2022

PdL
20 µM

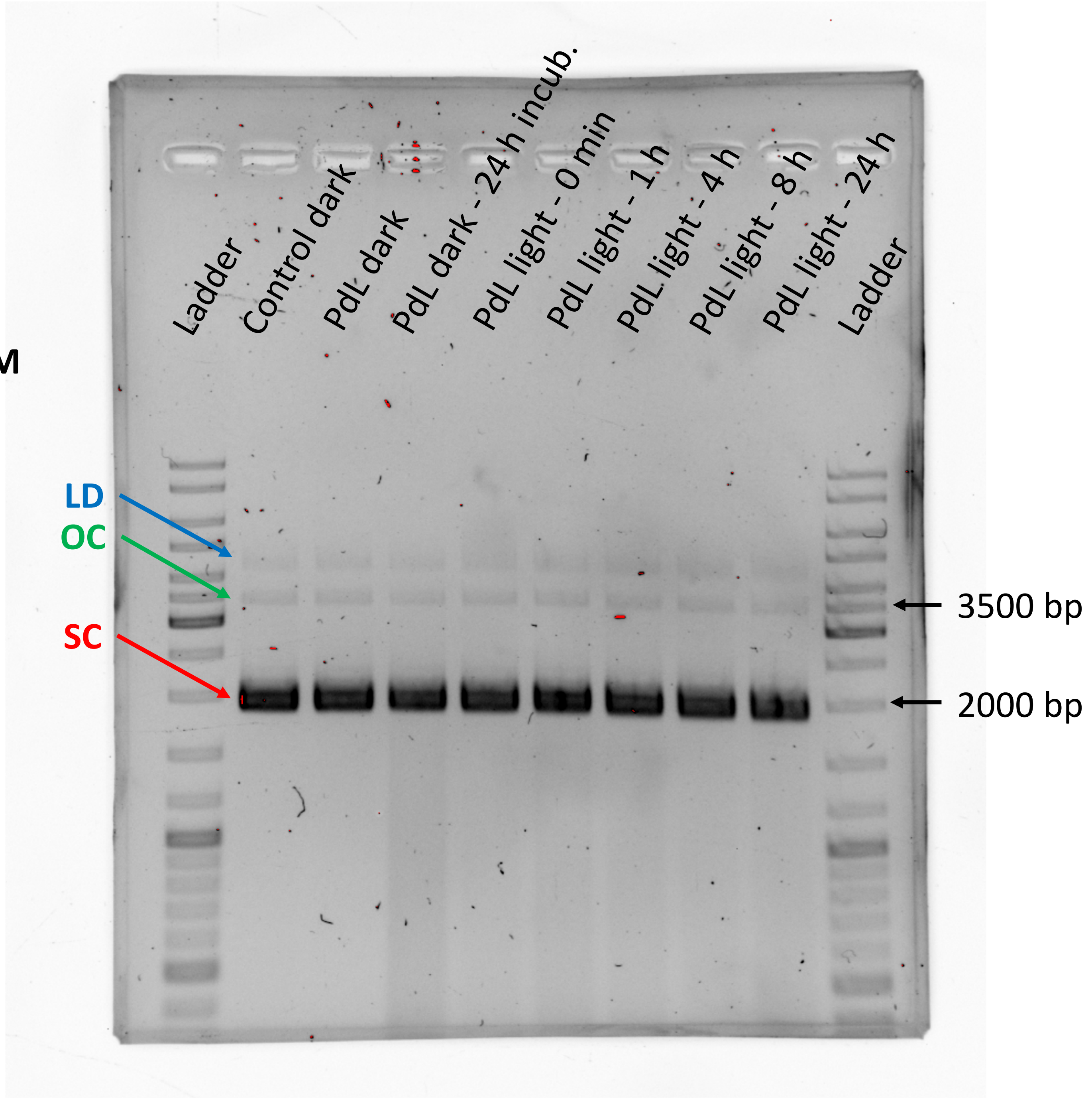

GeneRuler DNA Ladder Mix

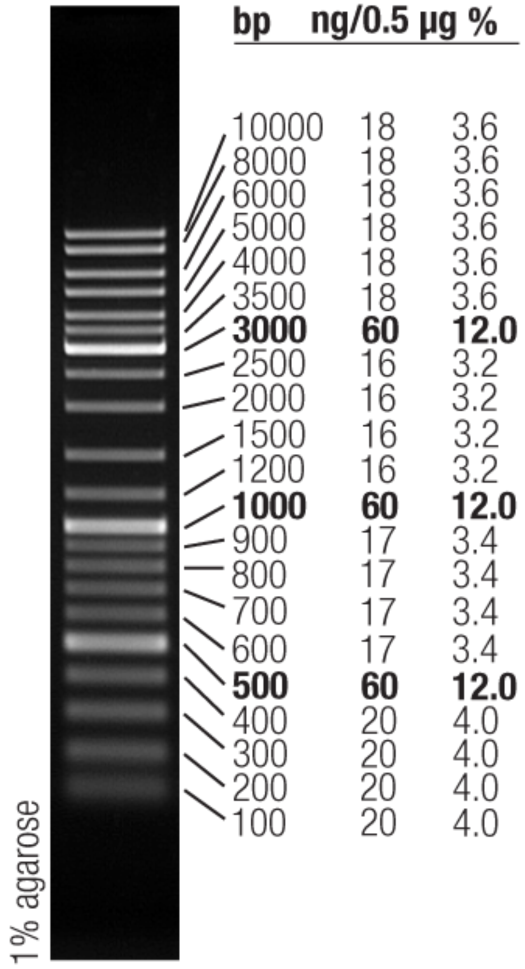

0.5 µg/lane, 8 cm length gel,
1X TAE, 7 V/cm, 45 min

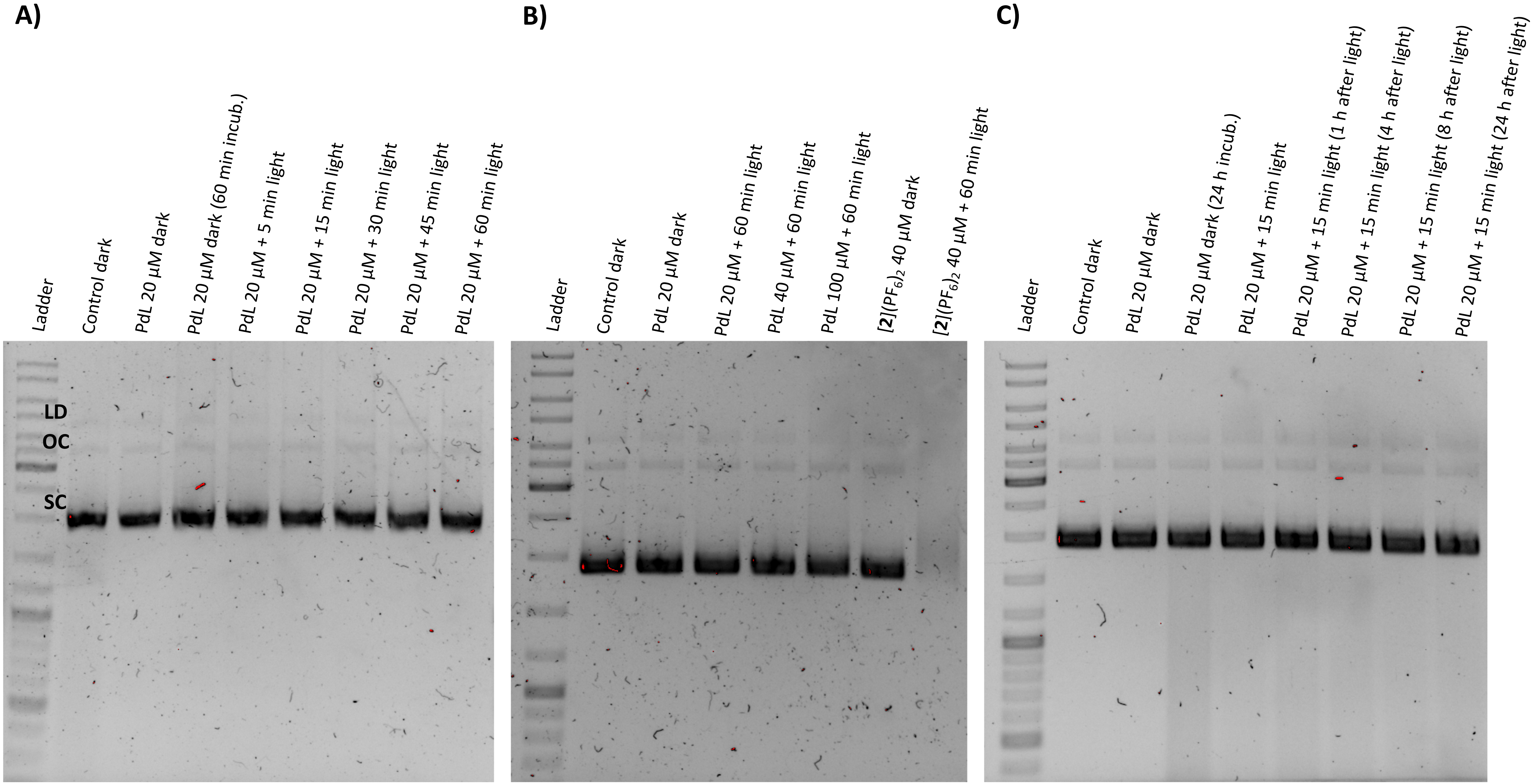
A)
Ladder
Control dark
PdL 20 μM dark
PdL 20 μM dark (60 min incub.)
PdL 20 μM + 5 min light
PdL 20 μM + 15 min light
PdL 20 μM + 30 min light
PdL 20 μM + 45 min light
PdL 20 μM + 60 min light
LD
OC
SC
B)
Ladder
Control dark
PdL 20 μM dark
PdL 20 μM + 60 min light
PdL 40 μM + 60 min light
PdL 100 μM + 60 min light
[2](PF6)2 40 μM dark
[2](PF6)2 40 μM + 60 min light
C)
Ladder
Control dark
PdL 20 μM dark
PdL 20 μM dark (24 h incub.)
PdL 20 μM + 15 min light
PdL 20 μM + 15 min light (1 h after light)
PdL 20 μM + 15 min light (4 h after light)
PdL 20 μM + 15 min light (8 h after light)
PdL 20 μM + 15 min light (24 h after light)
